# Supplementary material for: Demographic and genetic factors influence the abundance of infiltrating immune cells in human tissues
Source: Nat Commun. 2020 May 5;11:2213. doi: 10.1038/s41467-020-16097-9 (PMC7200670; doi:10.1038/s41467-020-16097-9)
Supplement: Supplementary file 1 — Supplementary Information [file 41467_2020_16097_MOESM1_ESM.pdf]

DEMOGRAPHIC AND GENETIC FACTORS INFLUENCE THE ABUNDANCE OF INFILTRATING IMMUNE CELLS IN HUMAN TISSUES

Marderstein et al.

SUPPLEMENTARY FIGURES

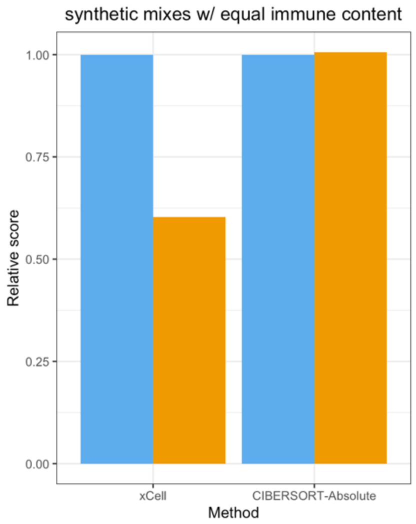

Supplementary Figure 1: Two synthetic samples with 10% CD4 T cell content. Sample 1 is in blue, sample 2 is in orange. CD4+ T cell content was estimated in both samples using xCell and CIBERSORT-Absolute. Along the y-axis, the relative difference in scores to sample 1 for each deconvolution method are shown: (Estimated CD4+ T cell score in Sample 1 / Estimated CD4+ T cell score in Sample *K*; therefore, sample 1 (blue) has a relative difference of 1).

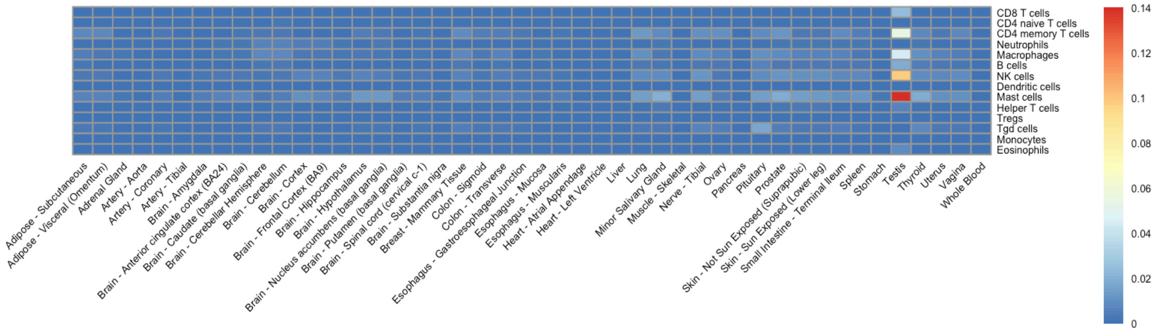

Supplementary Figure 2: Genes were shuffled within the GTEx gene expression profiles. For each signature gene (in the reference profile), we matched to another gene (via pan-tissue GTEx median gene expression values). We then replaced the gene expression values of the signature gene within the GTEx samples with the matched gene's expression values. We then performed deconvolution using the original CIBERSORT reference profile (LM22). Next, median CIBERSORT-Absolute scores for the 14 immune cell types were calculated within each tissue. Median values were visualized in a heatmap (sorted by tissues alphabetically). Nearly all scores were close to 0 with little variability between tissues and cell types.

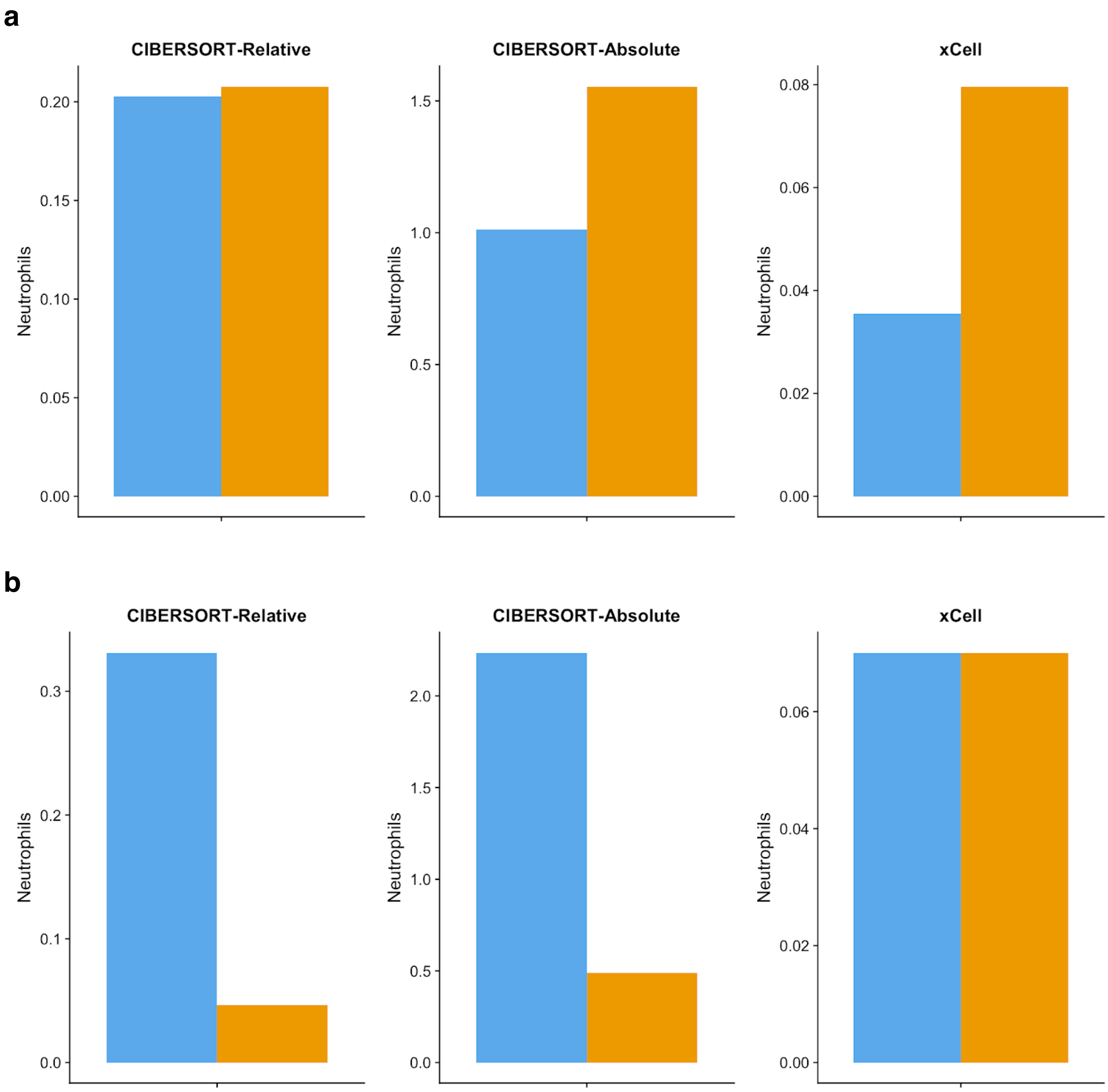

Supplementary Figure 3: Neutrophil content in lung tissue as estimated by CIBERSORT-Relative, CIBERSORT-Absolute, and xCell algorithms. (a) Individuals GTEX-14LLW (blue) and GTEX-17F96 (orange) have similar relative scores but different absolute scores. (b) Individuals GTEX-PLZ4 (blue) and GTEX-ZF2S (orange) have similar xCell scores, but very different CIBERSORT scores.

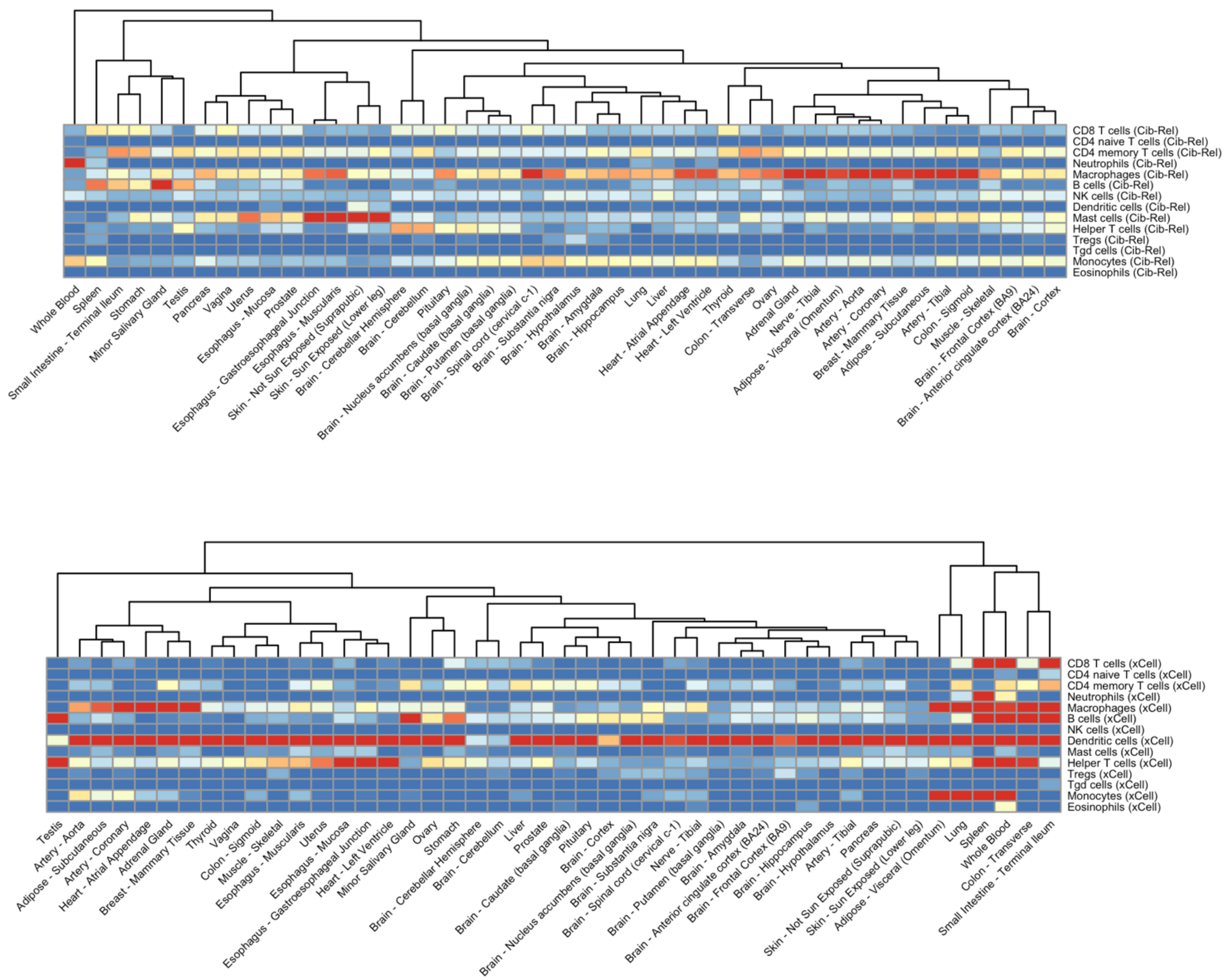

Supplementary Figure 4: Hierarchical clustering of GTEx tissues according to immune content, estimated by CIBERSORT-Relative (top) and xCell (bottom). Heatmap displays cell type median scores, with extremely large median score values (>0.3 for CIBERSORT-Relative, >0.05 for xCell) set to 0.3 for CIBERSORT-Relative and 0.05 for xCell.

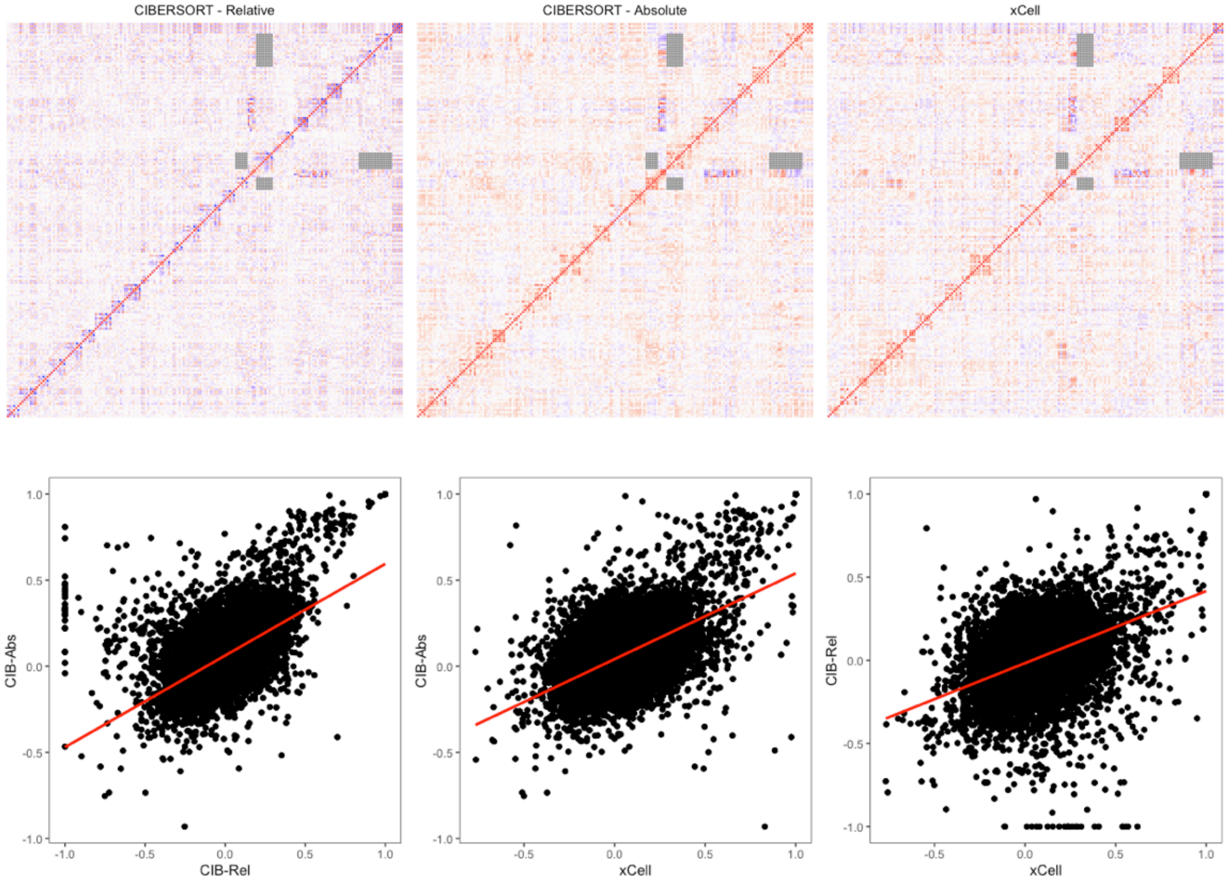

Supplementary Figure 5: Pairwise correlations measured between all 189 filtered infiltration phenotypes within each deconvolution method. *Top row*: Correlations are displayed via heatmap, which have been sorted by tissue. Each row and column is the same infiltration phenotype in each of the three plots. *Bottom row*: Correlations between infiltration phenotypes are compared between deconvolution methods. X-axis represents the set of correlations from one deconvolution method, y-axis represents the set of correlations from another deconvolution method. Red line shows the line of best fit.

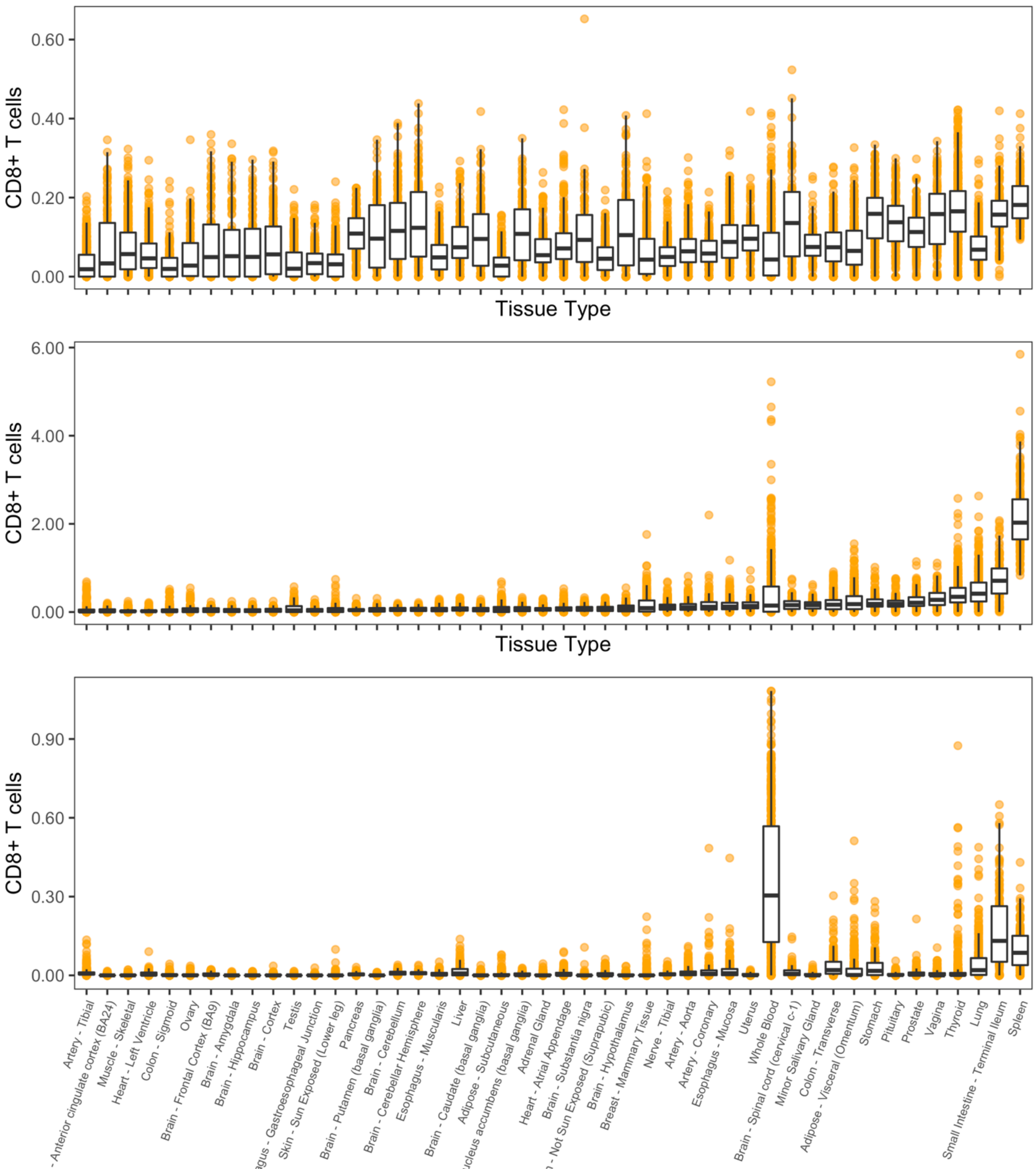

Supplementary Figure 6: Estimates of CD8+ T cell content in n=11141 samples across 46 GTEx tissues, segmented by each deconvolution method: CIBERSORT-Relative, then CIBERSORT-Absolute, and finally xCell. Tissues are sorted by median CIBERSORT - Absolute score. Data are summarized as boxplots where the middle line is the median, the lower and upper hinges represent the first and third quartiles, and the whiskers extend from the hinge with a length of 1.5 x the inter-quartile range. All data points are plot individually.

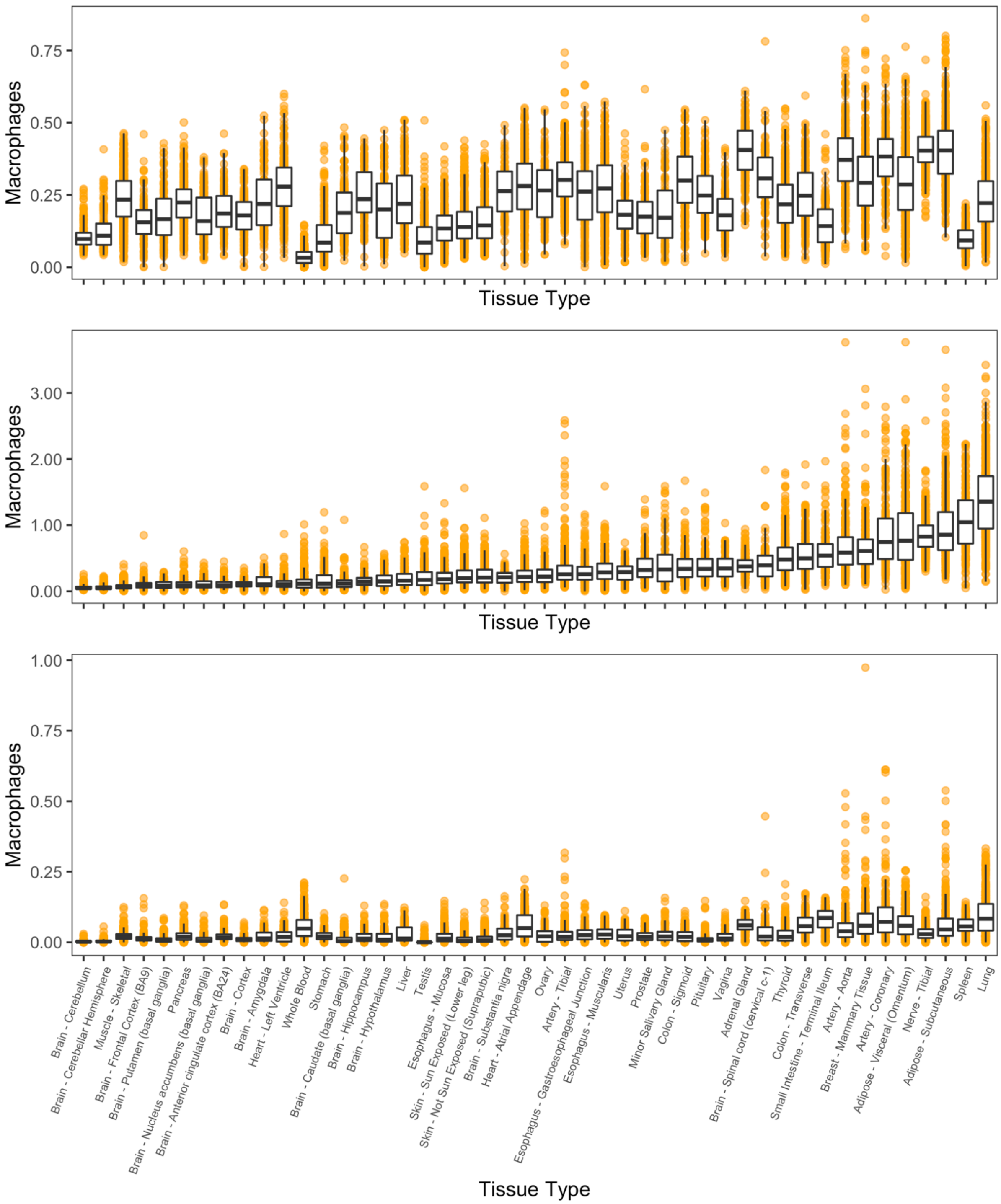

Supplementary Figure 7: Estimates of macrophages in n=11141 samples across 46 GTEx tissues, segmented by each deconvolution method: CIBERSORT-Relative, then CIBERSORT-Absolute, and finally xCell. Tissues are sorted by median CIBERSORT - Absolute score. Data are summarized as boxplots where the middle line is the median, the lower and upper hinges represent the first and third quartiles, and the whiskers extend from the hinge with a length of 1.5 x the inter-quartile range. All data points are plot individually.

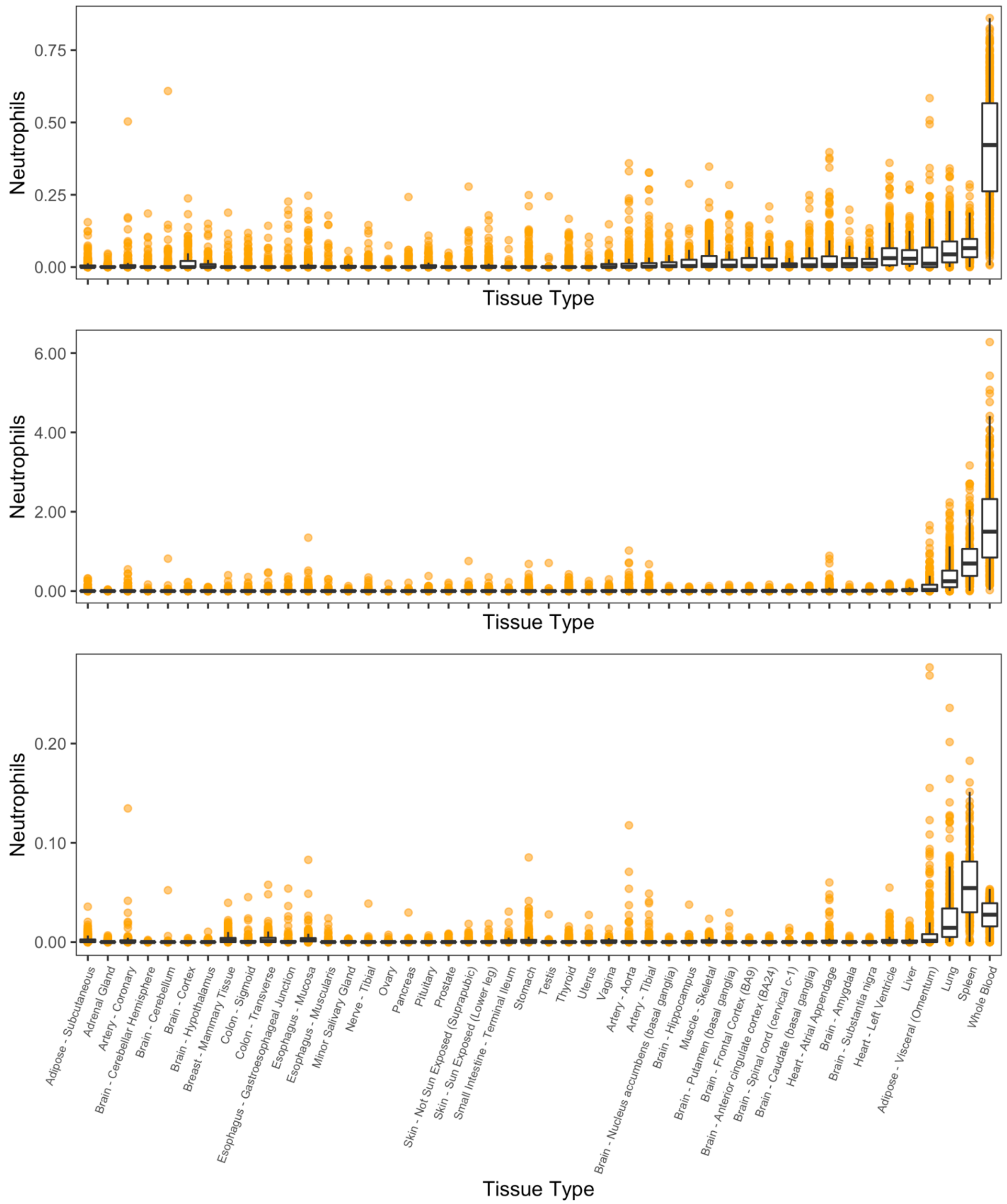

Supplementary Figure 8: Estimates of neutrophils in n=11141 samples across 46 GTEx tissues, segmented by each deconvolution method: CIBERSORT-Relative, then CIBERSORT-Absolute, and finally xCell. Tissues are sorted by median CIBERSORT - Absolute score. Data are summarized as boxplots where the middle line is the median, the lower and upper hinges represent the first and third quartiles, and the whiskers extend from the hinge with a length of 1.5 x the inter-quartile range. All data points are plot individually.

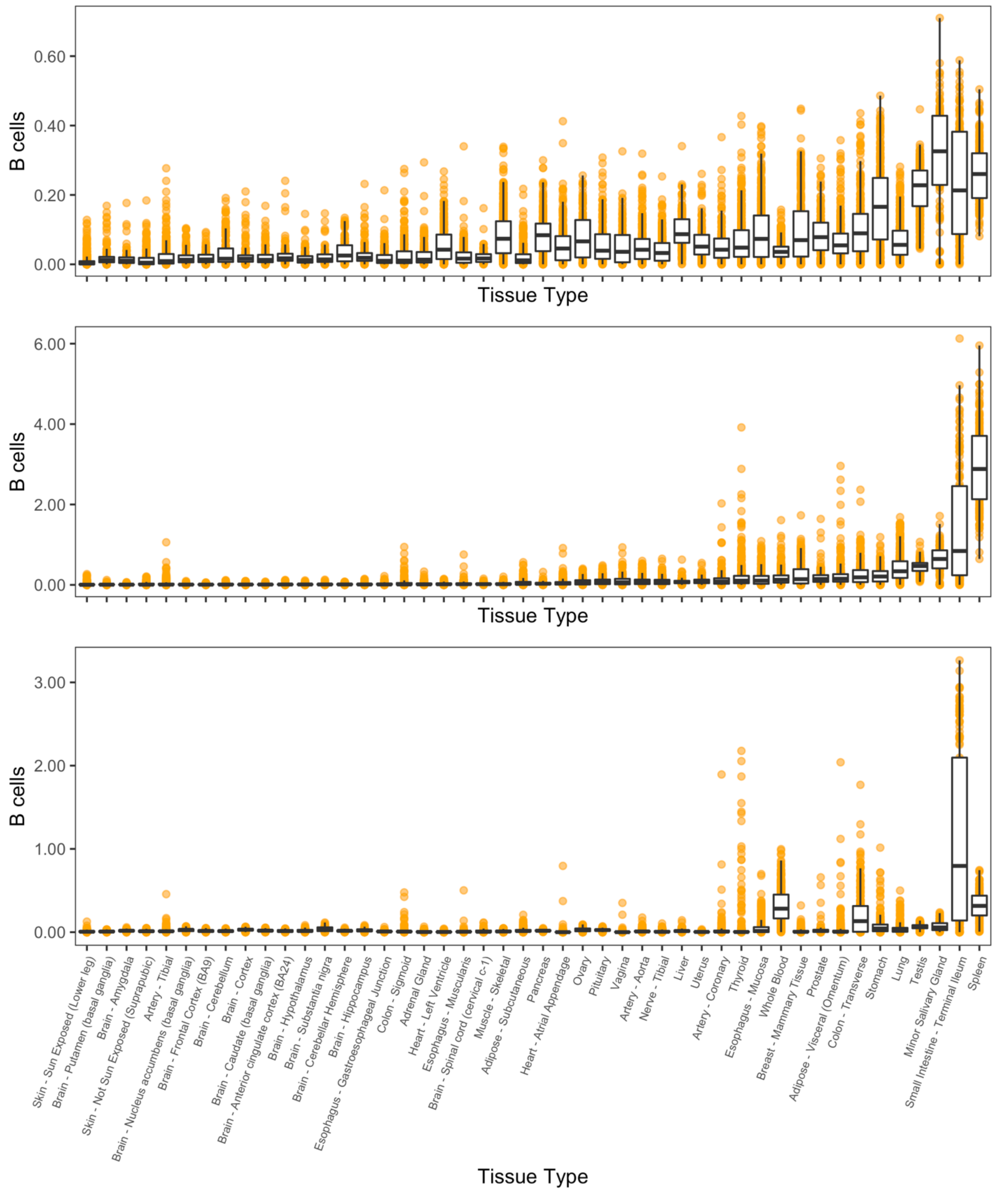

Supplementary Figure 9: Estimates of B cells in n=11141 samples across 46 GTEx tissues, segmented by each deconvolution method: CIBERSORT-Relative, then CIBERSORT-Absolute, and finally xCell. Tissues are sorted by median CIBERSORT - Absolute score. Data are summarized as boxplots where the middle line is the median, the lower and upper hinges represent the first and third quartiles, and the whiskers extend from the hinge with a length of 1.5 x the inter-quartile range. All data points are plot individually.

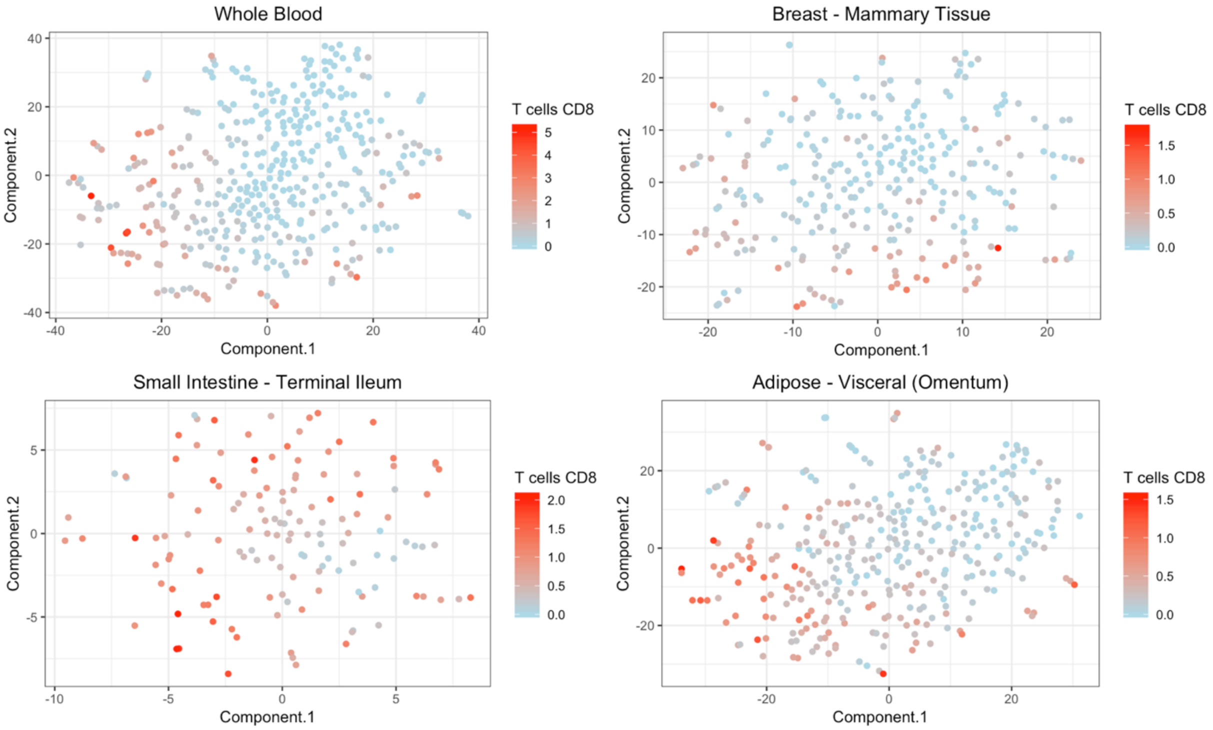

Supplementary Figure 10: t-SNE clustering of immune content within a single tissue type. t-SNE was performed on the 22 original immune cell type scores from CIBERSORT-Absolute deconvolution. Each point represents a unique sample from a different individual, which has been colored by the amount of measured CD8+ T cell content (low content: blue; high content: red).

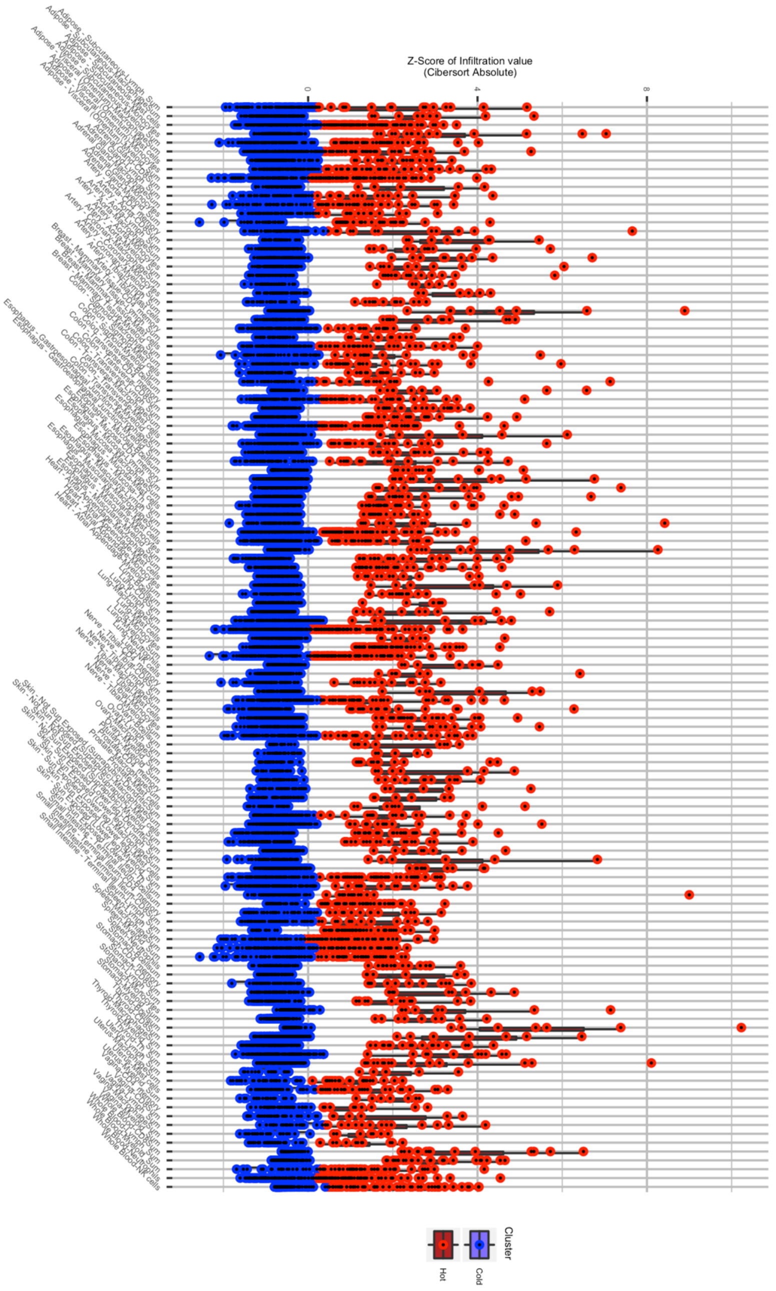

Supplementary Figure 11: “Hot” (red) and “cold” (blue) consensus clusters for n=6741 samples across 123 of the infiltration phenotypes where DEGs were identified. Immune cell type scores were converted into z-scores for visual purposes, such that all infiltration phenotypes could be visualized on the same scale (otherwise, the variation in phenotypes with lower mean and variances would be difficult to see). Data are summarized as boxplots where the middle line is the median, the lower and upper hinges represent the first and third quartiles, and the whiskers extend from the hinge with a length of 1.5 x the inter-quartile range. All data points are plot individually.

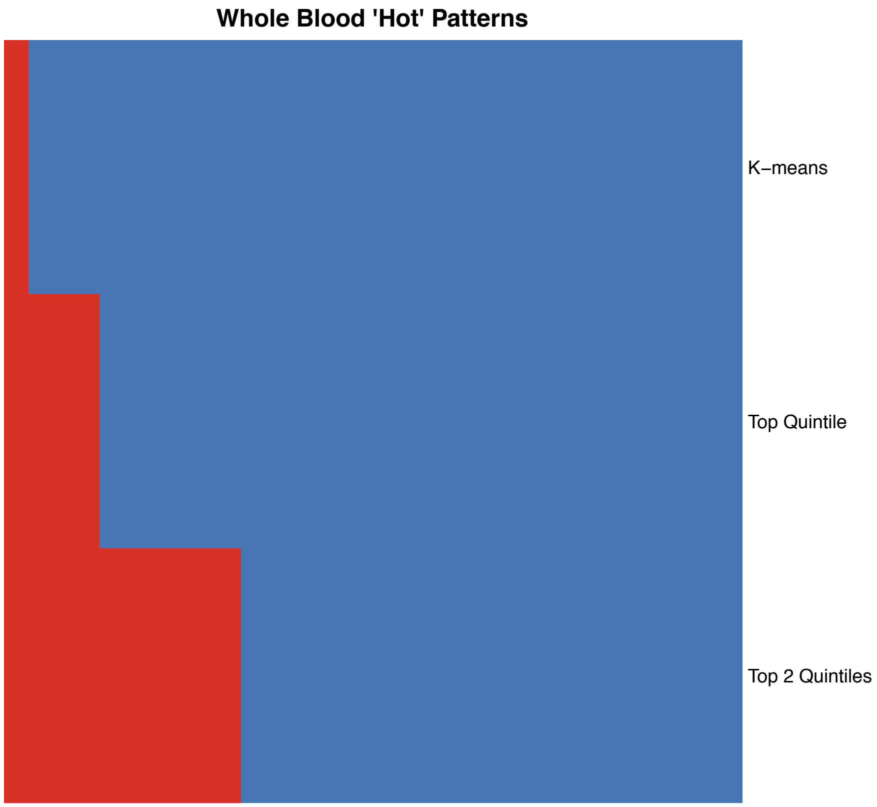

Supplementary Figure 12: “Hot” CD8 T cell cases across the whole blood samples, labelled by three different approaches: k-means consensus clustering, top quintile, and top two quintiles. Each column represents a different sample, while each row represents a different hot-labeling approach. Red cells indicate hot cases, while blue indicates not hot cases.

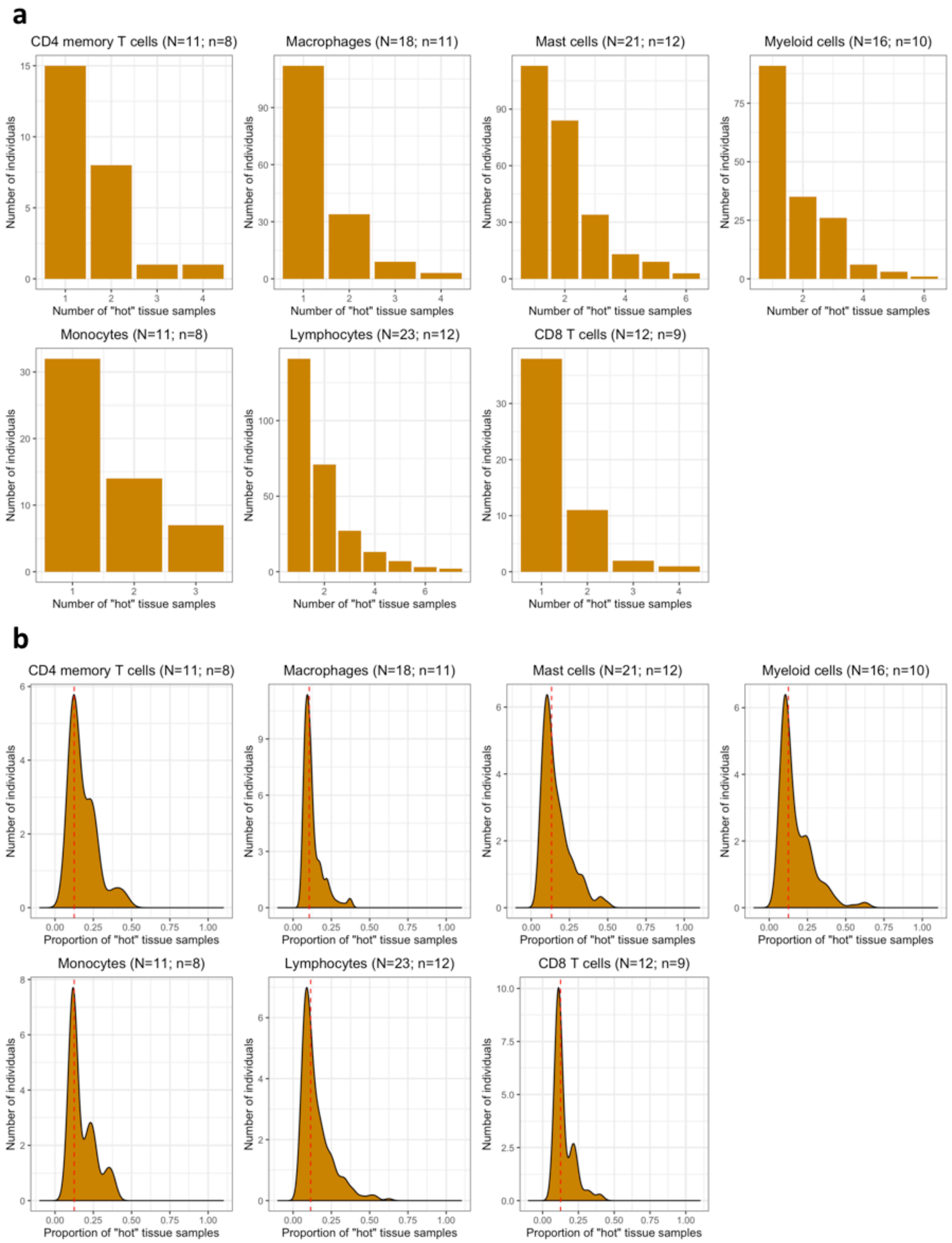

Supplementary Figure 13: Examination of “hot” patterns across tissues for a single individual, separated by cell type. Individuals were subset to those that are in at least “one” hot cluster of that cell type and contributed at least 8 tissues (post-filtering). *N* is number of total tissues for that cell type, and *n* is median number of contributed tissues from a single individual. Clusters are based on consensus clustering. (a) Histograms describing the number of tissues that each individual is in the “hot” cluster. (b) Density plot showing the proportion of tissues from a single individual that are labeled “hot”.

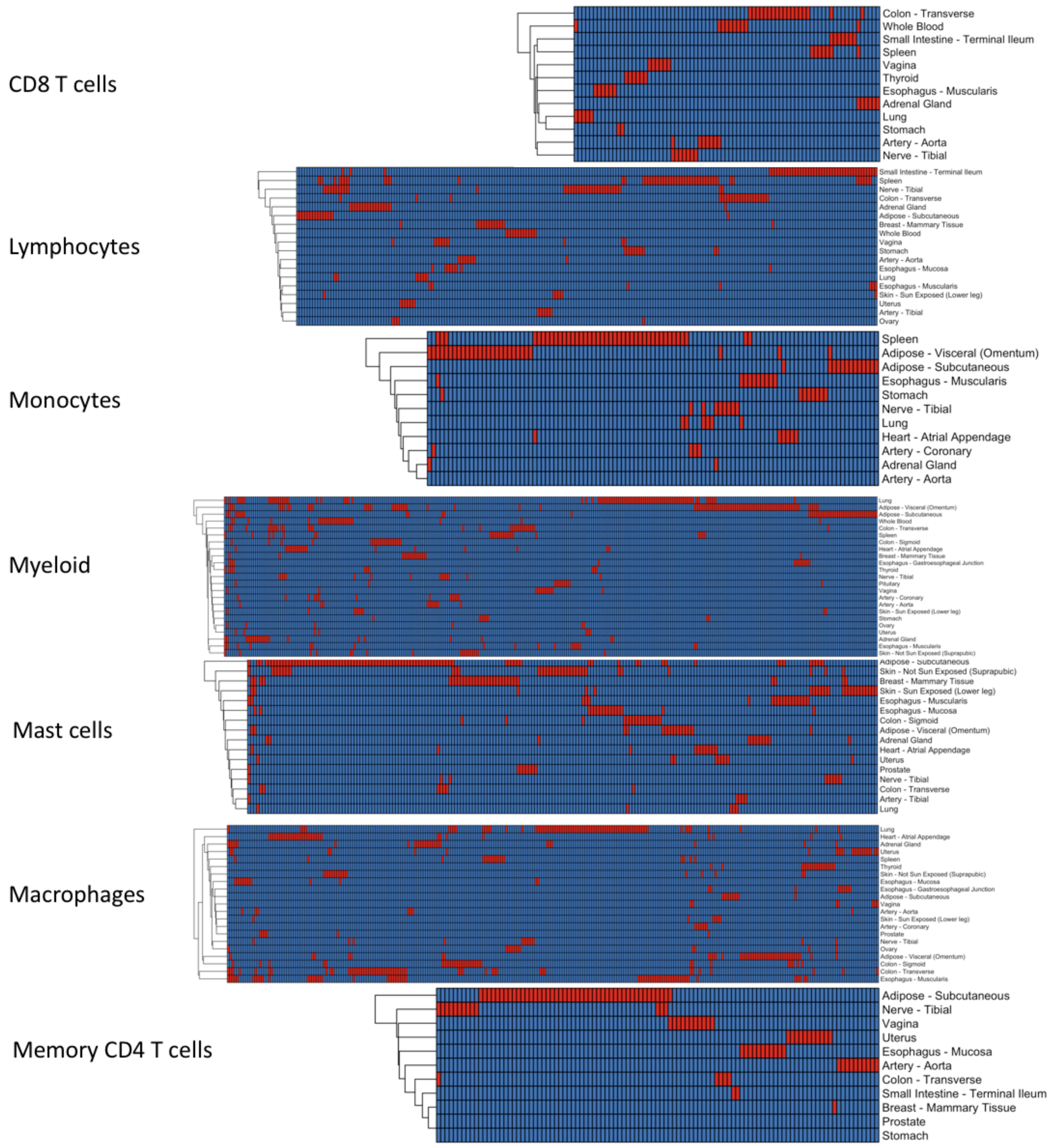

Supplementary Figure 14: Heatmaps display “hot” patterns across tissues and individuals. Clusters were determined by the consensus k-means clustering approach. Rows represent tissues and columns represent individuals. Red indicates the individual was labeled “hot” in that tissue type, while blue represents not “hot” (intermediate, cold, or missing data since an individual does not have a sample for every tissue type). Row and columns were clustered by Euclidean distance. In these figures, only tissues with > 6 hot and cold samples are shown.

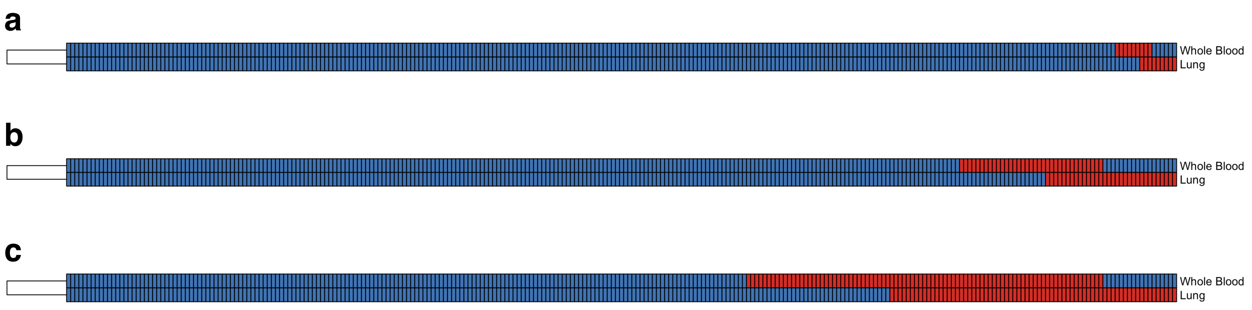

Supplementary Figure 15: Heatmaps display “hot” patterns across individuals with both lung and whole blood samples. If individuals do not have both whole blood and lung samples, individuals were removed from further analysis. “Hot” groupings were determined by (a) consensus k-means clustering, (b) quintiles, and (c) top two quintiles. Rows represent tissues and columns represent individuals. Red indicates the individual was labeled “hot” in that tissue type, while blue represents not “hot” (intermediate or cold). Columns (individuals) were clustered by Euclidean distance. Therefore, due to differences in clustering, one column across the three plots represents three different individuals.

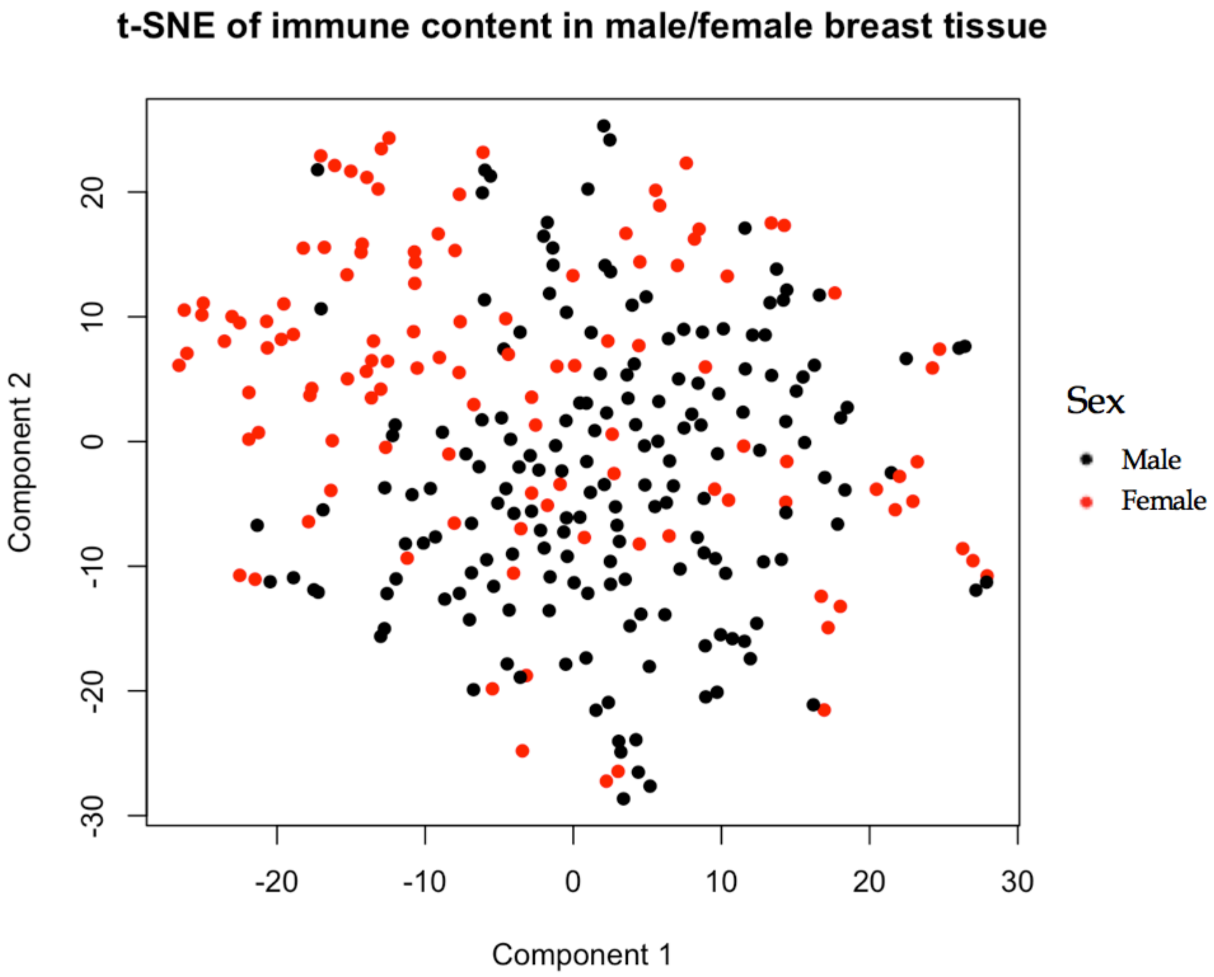

Supplementary Figure 16: t-SNE plot of immune content in breast tissue, calculated on the 22 immune cell type matrix from CIBERSORT - Absolute. Each point is a separate individual, colored by sex.

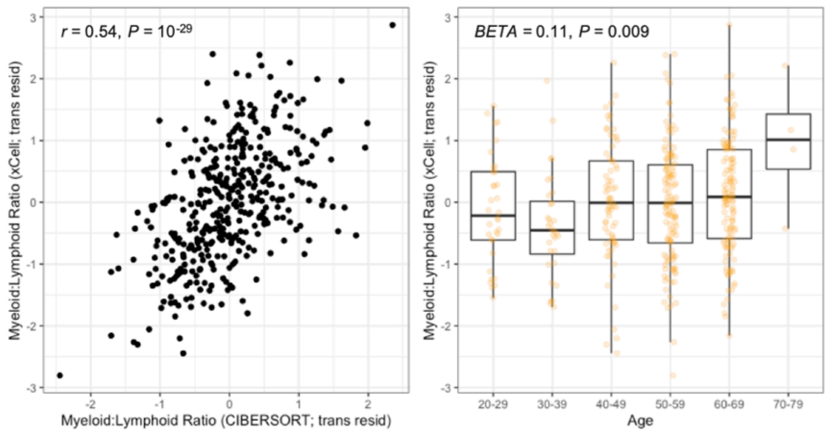

Supplementary Figure 17: Myeloid:Lymphoid ratios were calculated in n=407 whole blood samples and adjusted for linear model covariates, minus age (see **Methods)**. Left, the Myeloid:Lymphoid phenotype from xCell is plot against the phenotype derived from CIBERSORT. The Pearson product-moment correlation coefficient, *r*, is calculated between CIBERSORT and xCell, and p-value calculated using a t distribution. Right, the relationship between age and myeloid:lymphoid ratio in xCell. Beta represents the effect size from the linear model between age (numerical; discrete, binned into 10-year categories) and myeloid:lymphoid ratio, and the p-value represents the significance of the regression coefficient. Myeloid:lymphoid values are covariate-adjusted. Data is summarized as a boxplot where the middle line is the median, the lower and upper hinges represent the first and third quartiles, and the whiskers extend from the hinge with a length of 1.5 x the inter-quartile range. All data points are plot individually.

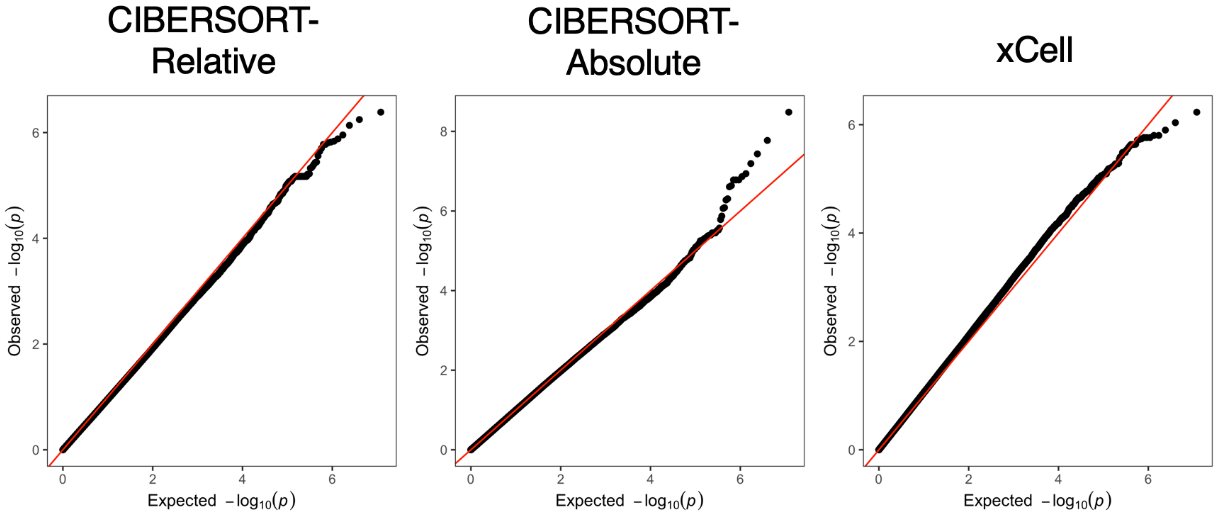

Supplementary Figure 18: 5+ million genome-wide genetic variants were tested for association with Helper T cells in thyroid samples. The SNP-association *P*-values in the separate analyses are visualized along the y-axis in a qq-plot, where the x-axis represents the expected -log10 *P*-values under the null distribution. Figure 5c shows the combined Empirical Brown's *P*-values from these 3 separate analyses. Each *P*-value is the coefficient significance of a SNP from a regression model on the helper T cell phenotype.

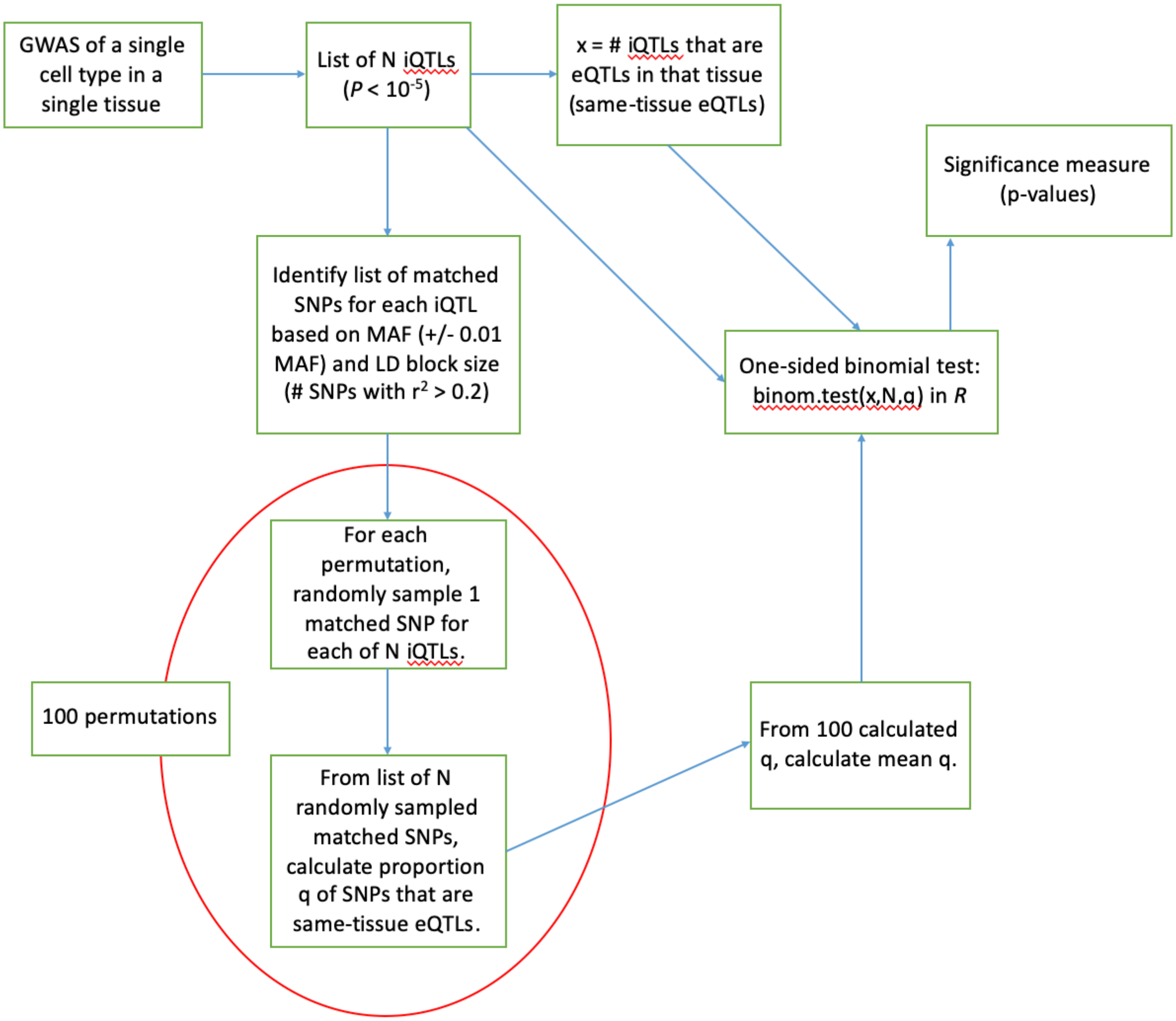

Supplementary Figure 19: Overview of the test for eQTL enrichment in iQTL findings. This describes the statistical method based on a two-sided binomial test.

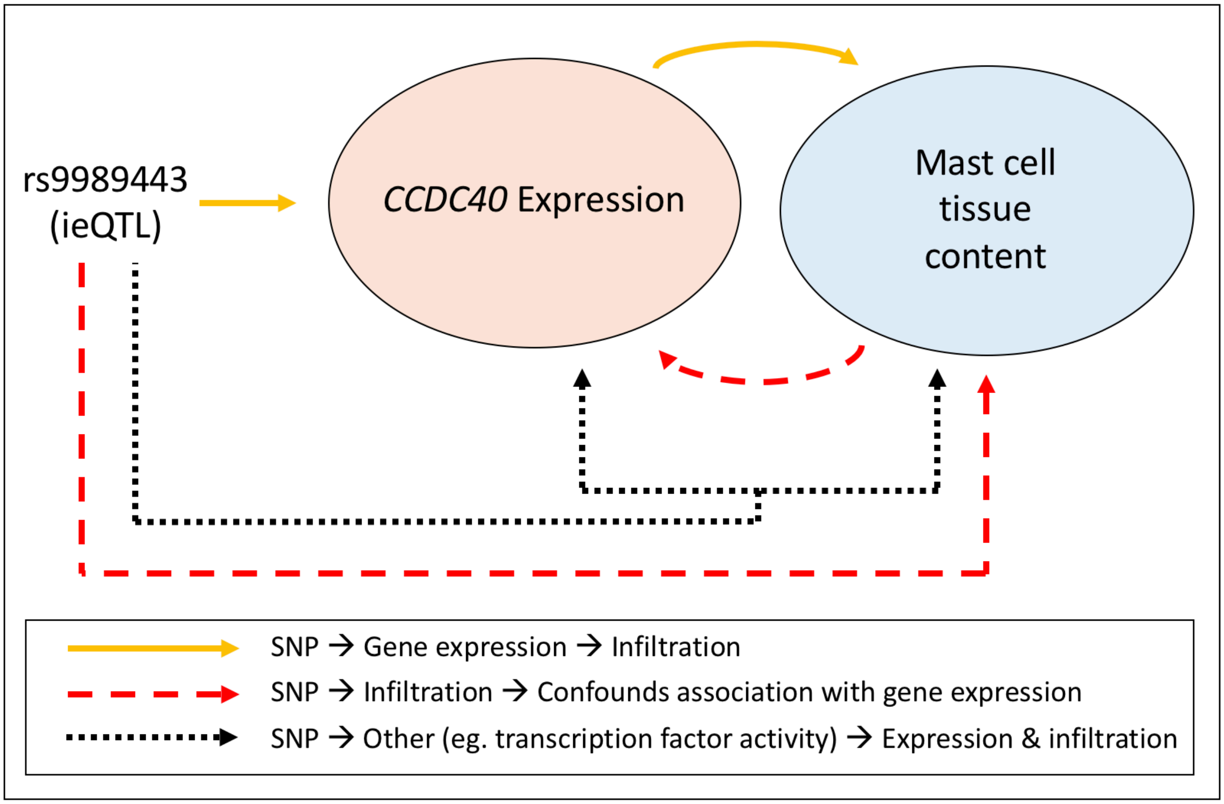

Supplementary Figure 20: ieQTL directionality is unclear**.** Example describing the potential relationships between rs9989443 and its association with both CCDC40 gene expression and mast cells in the esophagus muscularis samples.

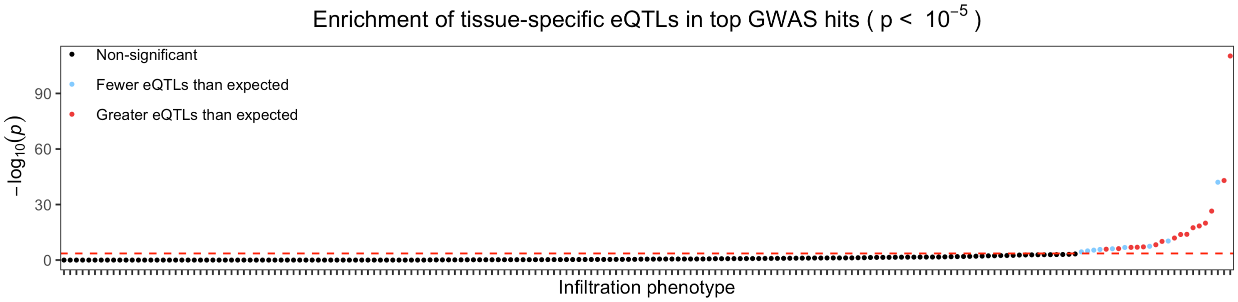

Supplementary Figure 21: eQTL enrichment in iQTL results using a two-sided binomial test, as described in Supplementary Figure 19. The y-axis describes the -log10 *P*-values from the test, and the x-axis describes each infiltration phenotype analyzed and sorted by significance.

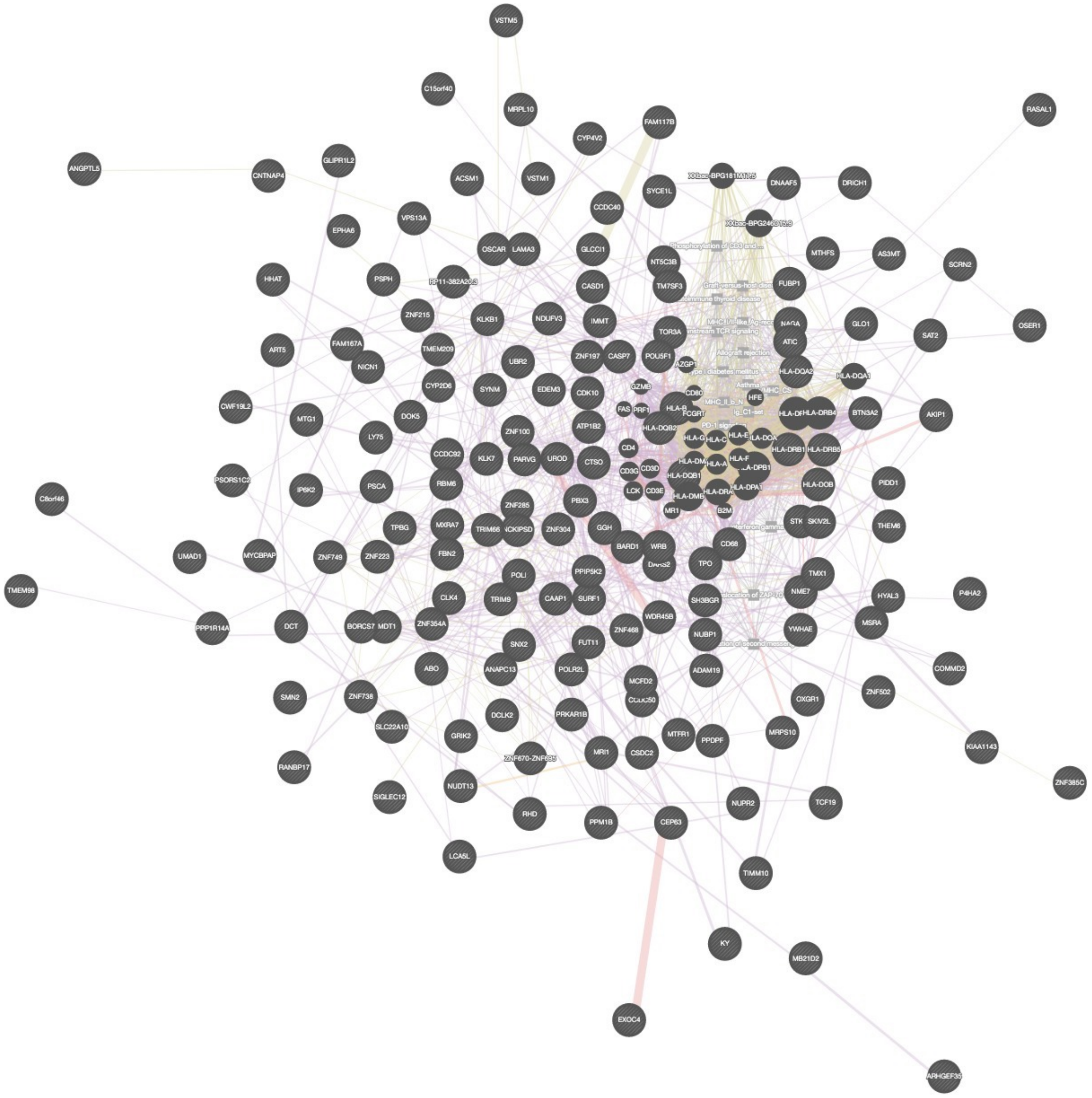

Supplementary Figure 22: GeneMania network of all ieGenes from ieQTLs that are associated with infiltration phenotypes at a relaxed $p < 10^{-5}$ threshold.

SUPPLEMENTARY TABLES

Cell Type	Scenario	CIBERSORT Mode	Correlation
CD4+ T cells	Tissue	Relative	0.8031571
CD4+ T cells	Tissue	Absolute	0.8986278
CD4+ T cells	Immune Cell	Relative	0.8460768
CD4+ T cells	Immune Cell	Absolute	0.8106775
CD8+ T cells	Tissue	Relative	0.6470465
CD8+ T cells	Tissue	Absolute	0.7115709
CD8+ T cells	Immune Cell	Relative	0.848441
CD8+ T cells	Immune Cell	Absolute	0.8001204

Supplementary Table 1: Correlation of estimated scores (from CIBERSORT) versus true quantity of cell type. True quantity of cell type measured as % of whole sample (absolute infiltration) and % of immune content (relative proportionality).

Cell Type	Scenario	xCell mode	Correlation
CD4+ T cells	Tissue	Tissue-by-tissue	0.9583721
CD4+ T cells	Immune Cell	Tissue-by-tissue	0.6517696
CD8+ T cells	Tissue	Tissue-by-tissue	0.9037872
CD8+ T cells	Immune Cell	Tissue-by-tissue	0.449362
CD4+ T cells	Tissue	Simultaneous	0.9214696
CD4+ T cells	Immune Cell	Simultaneous	0.6087092
CD8+ T cells	Tissue	Simultaneous	0.8419683
CD8+ T cells	Immune Cell	Simultaneous	0.4063541
CD4+ T cells*	Immune Cell*	Tissue-by-tissue*	0.7313011*
CD8+ T cells*	Immune Cell*	Tissue-by-tissue*	0.5955122*

Supplementary Table 2: Correlation of xCell scores with true amounts, as tested on the simulated synthetic mixes in the “tissue” scenario. xCell mode “Tissue-by-tissue” describes estimation on each tissue separately, while mode “Simultaneous” describes estimation on the full gene expression matrix (including different tissues at once). Default scores are not normalized (do not sum to 1). Last 2 rows (denoted by asterisk) represent performance of xCell in the “immune cell” scenario when the xCell scores were normalized.

Cell type phenotypes	xCell phenotypes used	CIBERSORT phenotypes used
CD8+ T cells	"CD8+ naive T-cells", "CD8+ T-cells", “CD8+ Tcm”, “CD8+ Tem”	“T cells CD8”
CD4+ naïve T cells	"CD4+ naive T-cells”	“T cells CD4 naïve”
CD4+ memory T cells	“CD4+ Tcm”, “CD4+ Tem”, “CD4+ memory T-cells”	“T cells CD4 memory activated”, “T cells CD4 memory resting”
Helper T cells	“Th1 cells”, “Th2 cells”	“T cells follicular helper”
Regulatory T cells	“Tregs”	“T cells regulatory (Tregs)”
Gamma delta T cells	“Tgd cells”	“T cells gamma delta”
B cells	"B-cells", "Class-switched memory B-cells", "Memory B-cells", "naive B-cells", "pro B-cells", "Plasma cells"	“B cells naive”, “B cells memory”, “Plasma cells”
NK cells	“NK cells”	“NK cells activated”, “NK cells resting”
Neutrophils	“Neutrophils”	“Neutrophils”
Macrophages	“Macrophages”, “Macrophages M1”, “Macrophages M2”	“Macrophages M0”, “Macrophages M1”, “Macrophages M2”
Dendritic cells	“aDC”, “cDC”, “DC”, “iDC”, “pDC”	“Dendritic cells resting”, “Dendritic cells activated”
Mast cells	“Mast cells”	“Mast cells resting”, “Mast cells activated”
Monocytes	“Monocytes”	“Monocytes”
Eosinophils	“Eosinophils”	“Eosinophils”

Supplementary Table 3: Merging of reference cell types to create the specific immune cell type phenotype scores used in the research study.

**Cell type phenotypes**	**xCell phenotypes used**	**CIBERSORT phenotypes used**
Lymphocytes	"CD4+ memory T-cells", "CD4+ naive T-cells", "CD4+ T-cells", "CD4+ Tcm", "CD4+ Tem", "CD8+ naive T-cells", "CD8+ T-cells", "CD8+ Tcm", "CD8+ Tem", "NKT", "Tgd cells", "Th1 cells", "Th2cells", "Tregs", “NK cells”, "B-cells", "Class-switched memory B-cells", "Memory B-cells", "naive B-cells", "pro B-cells", "Plasma cells"	"B cells naive", "B cells memory", "Plasma cells", "T cells CD8", "T cells CD4 naive", "T cells CD4 memory resting", "T cells CD4 memory activated", "T cells follicular helper", "T cells regulatory (Tregs)", "T cells gamma delta", "NK cells resting", "NK cells activated"
Myeloid cells	“Macrophages”, “Macrophages M1”, “Macrophages M2”, “aDC”, “cDC”, “DC”, “iDC”, “pDC”, “Mast cells”, “Monocytes”, “Eosinophils”, “Basophils”, “Neutrophils”	“Neutrophils”, “Macrophages M0”, “Macrophages M1”, “Macrophages M2”, “Dendritic cells resting”, “Dendritic cells activated”, “Mast cells resting”, “Mast cells activated”, “Monocytes”, “Eosinophils”

Supplementary Table 4: Merging of reference cell types to create the broader immune cell type phenotype scores used in the research study.

Tissue 1	Tissue 2	Cell	P-value
Artery - Aorta	Lung	Mast cells	5.00E-06
Esophagus - Gastroesophageal Junction	Esophagus - Muscularis	Mast cells	1.08E-07
Esophagus - Mucosa	Esophagus - Muscularis	Mast cells	2.08E-05
Adipose - Visceral (Omentum)	Colon - Sigmoid	Monocytes	5.83E-08
Lung	Whole Blood	Lymphocytes	2.63E-09
Adipose - Visceral (Omentum)	Nerve - Tibial	CD8 T cells	1.93E-05
Adipose - Visceral (Omentum)	Whole Blood	CD8 T cells	1.09E-09
Lung	Whole Blood	CD8 T cells	1.11E-17
Thyroid	Whole Blood	CD8 T cells	1.04E-06

Supplementary Table 5: Significant results from assessing the sharing of "hot" patterns between two tissues. Clusters are based on the top two quintiles (40%) approach, and *P*-values are based on Fisher's exact *P*-values.

Gene IDs
ENSG00000173295
ENSG00000272733
ENSG00000189269
ENSG00000129055
ENSG00000182923
ENSG00000240053
ENSG00000143156

Supplementary Table 6: Target ieGenes of ieQTLs ($p < 10^{-5}$) in multiple infiltration phenotypes.

SUPPLEMENTARY NOTES

Supplementary Note 1: Theoretical comparison of relative versus absolute scores

We motivate incorporating relative and absolute outputs from CIBERSORT into analyses by describing how both focus on capturing separate aspects of cellular heterogeneity.

Let x be the true % of the sample that is immune cell type x.
Let y be the true % of the sample that is immune cell type y.
Let z be the total % of the sample represented by immune infiltration, $x + y$.

Let there be three different samples with infiltration: (x_1,y_1), (x_2,y_2), (x_3,y_3) = (10,0), (10,10), (20,0). Therefore, (z_1, z_2, z_3) = (10, 20, 20).

Let κ be the relative percentage (%) of cell type x to the infiltration profile. This can be described generally by the following expectation:

(Eq. 1)

$$E[\kappa] = x/z$$

CIBERSORT-Relative measures the relative % of a cell type to the other cell types in its reference (e.g. LM22). For example, CIBERSORT-Relative would directly estimate κ for immune cell type x. Using Equation 1, $(E[\kappa_1], E[\kappa_2], E[\kappa_3])$ = (1, 0.5, 1). CIBERSORT-Absolute scales this relative score to reflect the amount of overall infiltration. Let π represent this scaling factor. For the purpose of this example, we allow:

(Eq. 2)

$$E[\pi] = z/\min(z_1, z_2, z_3)$$

such that (π_1, π_2, π_3) = (1, 2, 2). Let γ be the CIBERSORT-Absolute score, which should follow the below expectation:

(Eq. 3)

$$E[\gamma] = E[\pi]E[\kappa]$$

Using Equation 3, we can calculate:

$$E[\gamma_1] = E[\pi_1]E[\kappa_1] = (1)(1) = 1$$
$$E[\gamma_2] = \cdots = (2)(0.5) = 1$$
$$E[\gamma_3] = \cdots = (2)(1) = 2$$

Now, $E[\gamma_1] = E[\gamma_2] < E[\gamma_3]$ while $E[\kappa_2] < E[\kappa_1] = E[\kappa_3]$. Therefore, absolute and relative scores are quantifying different measures of the immune infiltration: relative proportions (%) of immune cells versus absolute counts of immune cells.

Furthermore, CIBERSORT-Relative calculates a better measure of the relative composition when $\pi_1 = \pi_2 = \pi_3$. This is achieved by not including the scaling factor π:

(Eq. 4)

$$Var[\gamma] = Var[\pi\kappa] > Var[\kappa]$$

Constant π reduces the variance of the output, since π typically has non-zero variance between samples.

Supplementary Note 2: Analyzing the utilization of multiple deconvolution methods in simulations

In the main text, we describe how xCell and CIBERSRT calculate scores that correlate with the true amounts and correlate with each other, but do not perfectly correlate with each other. In Supplementary Figure 1, we show how even in simulated synthetic mixes where the true amounts are known to be equal, the immune cell estimates can differ. For example, in this particular scenario, CIBERSORT-Absolute correctly detects that CD4+ T cell content is equal between the two simulated samples. In contrast, xCell produces an estimate for Sample 2 that is roughly 60% of Sample 1. Therefore, the overall correlation results in our simulations have shown that these methods describe alternative yet reasonably accurate perspectives of immune cells in the test sample; but, there are clear cases where there exist differences. These differences can lead to *effect size heterogeneity*, where an effect may be better detected in one deconvolution method compared to another, such as xCell versus CIBERSORT (due to biases from different algorithms or reference matrices) or Relative versus Absolute (does the genetic effect alter the composition of immune cells in the sample or does the genetic effect alter the total amount of a particular immune cell in the sample?). To identify associations, we hypothesized that it makes sense to utilize this statistical heterogeneity within analyses by leveraging shared signals across deconvolutions rather than ignoring it by choosing a single deconvolution method.

To test this hypothesis using our previously simulated *N*=80 synthetic samples, we consider a SNP analysis. We simulated a SNP genotype with MAF = 0.4 in N=80 individuals, coded as 0, 1, or 2 from a binomial distribution. In the causal scenario, we let the SNP randomly explain 0 – 8% variance in each of the three deconvolution phenotypes (CIBERSORT-Abs, CIBERSORT-Rel, and xCell) by (1) randomly selecting three values from a uniform distribution between 0 and 0.08 to allow effect size heterogeneity across the deconvolution outputs, and (2) rescaling the genotypic (binomial) and environmental (original deconvolution score) components. In the non-causal scenario, the SNP contributes 0% variance to each of the deconvolution phenotypes (thus, phenotypes used are the original deconvolution scores). We then tested the association between the SNP and phenotype for each deconvolution method using a linear model, merged these *P*-values using the combined Empirical Brown's method, and rejected the null hypothesis of no association when $P < 0.05$. We repeated this 10,000 times in the causal scenario and 10,000 times in the non-causal scenario.

We found that the power in the causal scenarios was 0.5164 using the Empirical Brown's method, but 0.4585, 0.4664, and 0.4662 in the xCell, CIBERSORT-Rel, and CIBERSORT-Abs separate analyses. Furthermore, we found that the false positive rate increase in the non-causal scenario was negligible: 0.0555 using the Empirical Brown's method, and 0.0517, 0.0509, and 0.0500 in the xCell, CIBERSORT-Rel, and CIBERSORT-Abs separate analyses. Finally, our analyses found zero non-causal SNPs with $P < 5 \times 10^{-8}$, but 5 of 10,000 using the EBM *P*-values, compared to 3, 1, and 2 in the separate analyses from xCell, CIBERSORT-Rel, and CIBERSORT-Abs. Therefore, the combined approach using Empirical Brown's method revealed superior power while maintaining low false positive rates as compared to the separate analyses.

Supplementary Note 3: Influence of random genes on deconvolution estimates as a control

When performing CIBERSORT deconvolution, we relied on an author-provided reference matrix (LM22). This matrix consists of specific sets of signature genes that differentiate the immune cell types. As a result, we could not swap the signature genes with non-signature genes within the CIBERSORT LM22 reference profile to test the influence of signature genes on deconvolution.

Instead, we shuffled the genes within the GTEx gene expression profiles. For each signature gene (in the reference profile), we matched to another gene (via pan-tissue GTEx median gene expression values). We then replaced the gene expression values of the signature gene within the GTEx samples with the matched gene's expression values. We then performed deconvolution using the original CIBERSORT reference profile (LM22).

Overall, the "shuffled" deconvolutions results had low variability and poor detection of immune cell types. CIBERSORT computes a *P*-value which tests the null hypothesis that no cell types from the reference profile (22 different immune cell types) are in the sample. In the actual (original) GTEx expression profiles, 2866 of 11141 samples had CIBERSORT *P*-values < 0.05. In contrast, only 4 of the 11141 shuffled GTEx expression profiles had CIBERSORT *P*-values < 0.05. This first observation describes how CIBERSORT did not identify conclusive immune cell concentrations in nearly all shuffled GTEx expression profiles, in stark contrast to the original GTEx data. Second, heatmaps of median CIBERSORT-Absolute scores across tissues (similar to Figure 2a) in shuffled GTEx expression profiles show low variability and low values across tissue types (Supplementary Figure 2). (The high amounts of some cell types in GTEx testis tissue can be explained by vastly different expression of some genes in GTEx testis tissue compared to other tissues, which might now be used as the "signature genes" for deconvolution.) Lastly, none of the inferred immune cell types will pass our infiltration phenotype filtering procedure described in the procedure. Therefore, the shuffled GTEx profiles result in 0 infiltration phenotypes. These observations suggest that the actual gene expression profiles result in infiltration results that are distinct from the shuffled expression profiles, and thus the shuffled expression profiles should be discarded from further analysis.

Supplementary Note 4: Deconvoluting GTEx expression profile and comparing methods

We first analyzed the reliability of cell type estimation between methods and noted that mean cell scores for immune cell types of interest demonstrated marked heterogeneity between deconvolution methods. For example, relative scores could be very similar but the absolute scores very different due to a higher amount of total infiltration (Supplementary Figure 3a). Individuals GTEX-14LLW and GTEX-17F96 had high proportions of neutrophil content in lung tissue according to CIBERSORT-Relative. However, a ~50% larger scaling factor for GTEX-17F96 resulted in different CIBERSORT-Absolute scores. xCell had even larger differences between the two individuals, with over twice as high estimated neutrophil content in GTEX-17F96. Similarly, xCell and CIBERSORT may also compute contrasting results for a single sample (Supplementary Figure 3b). But despite these case differences, we found that clustering of tissues by median scores were relatively consistent across each deconvolution method (Figure 2a, Supplementary Figure 4). In our hierarchical clustering across the three cell type scoring methods, many brain tissues cluster together, as well as the tissue pairings of sun exposed and unexposed skin tissue and the tissue pairings of coronary and aorta arteries. For many other tissues, the nearest-neighbor pairing was not exact across the 3 deconvolution methods but demonstrated concordance through consistent clustering along the same branches, such as vagina and uterus tissues from the female urogenital tract or the heart tissues. In contrast, there were several tissues without consistent pairings in nearest-neighbor or overall branch, such as lung and liver, which reflect differences between the cell type estimation methods. We noticed different clustering among sigmoid and transverse colon tissue. Transverse colon had a much greater immune presence compared to sigmoid colon samples (scaling factor from CIBERSORT - Absolute: 1.96 in transverse, 1.08 in sigmoid colon samples), with greater detection of CD8+ T cell content (and insignificant correlation of CD8+ T cells between sigmoid and transverse tissue samples from the same individual).

Furthermore, we examined the conservation of relationships between deconvoluted immune cell abundances across methods. First, we calculated pairwise correlation between all 189 infiltration phenotypes (see **Methods**) and found that pairwise correlations were well-correlated with pairwise correlations from another deconvolution method (Supplementary Figure 5). This suggests that observed relationships between tissue immune content abundances are generally consistent regardless of deconvolution method choice. Additionally, infiltration driven by the absolute amount of immune content is better captured in absolute outputs, as seen by more positive correlations across infiltration phenotypes in absolute scores compared to relative scores (Supplementary Figure 5). The mean value of pairwise correlation coefficients is $r = 0.0026$ for CIBERSORT-Relative scores, compared to $r = 0.064$ and $r = 0.044$ for CIBERSORT-Absolute scores and xCell scores. From our comparative analysis of deconvolution methods in GTEx, we were encouraged to try and use all three methods given the differences but general similarities.

Supplementary Note 5: Analysis of inter-tissue differences across GTEx tissues

We observed the highest neutrophil scores in whole blood, spleen, and lung tissues across each deconvolution method (Supplementary Figure 8). While neutrophils dominate whole blood content, and therefore are expected in high frequencies within spleen tissue, neutrophils are frequently observed in lung tissue inflammation[1]. We also find the small intestine samples from the terminal ileum to have high CD8+ T cell content (Supplementary Figure 6), which makes sense given the tissue's key role in digestion and encountering foreign microorganisms and possible infection. This also matches a gradient of increasing CD8+ T cell content from the rectum to further down the gastrointestinal tract (transverse and sigmoid colon), which corroborates previous clinical observations[2] and our own findings (Supplementary Figure 6).

Supplementary Note 6: Differential expression between inflamed and non-inflamed

Overall, we identified DEGs passing our statistical thresholds for 123 of the 130 tested infiltration phenotypes (log FC >= 2.0, FDR < 0.01) (see **Methods** and Figure 3b). Across phenotypes, we expected and found that the most common DEGs consisted of well-known markers of the corresponding immune cell types (Supplementary Data 5). For example, the most consistent DEGs across macrophage-hot clusters are macrophage markers utilized by the xCell and CIBERSORT algorithms for estimating macrophage content: *C1QB* (18/21 tissues), *VSIG4* (17/21), *MARCO* (17/21), and *CD163* (16/21). Interestingly, the most common DEGs not present in the deconvolution reference gene sets includes *C1QC* (16/21) and *FCGR3A* (16/21), which correspond to complement component and immunoglobulin Fc receptor, and have well-characterized roles in opsonization[22,23]. We then used the DEGs and Ingenuity Pathway Analysis (IPA) to identify dysregulated pathways, discover key upstream regulators, and find central disease and function ontologies (Supplementary Data 6-8). In our macrophage phenotypes, the most commonly dysregulated pathways were *TREM1* signaling, which is an amplifier of macrophages inflammation[24], cAMP-mediated signaling, and neuroinflammation and cardiac hypertrophy signaling (Supplementary Data 6). The most frequent upstream regulator predicted to be activated by IPA in the macrophage-hot clusters was *TGM2*, while *TFRC* (transferrin receptor) was the most commonly inhibited as predicted by IPA (Supplementary Data 7). The latter finding may be linked to the role of macrophages in sequestering iron during inflammatory states[25]. Disease and function ontologies indicated that the most commonly activated pathways in macrophage-hot samples were associated with leukocyte and lymphocyte migration (Supplementary Data 8). IPA on filtered gene sets implicated dysregulation of the LXR/RXR metabolic pathway in hot samples, with IL1 and IL6 as predicted activated upstream regulators, and ontologies related to cellular movement and phagocytosis.

Of the 10 CD8+ T-cell infiltration phenotypes, the most common DEGs in hot clusters are the CD8 lymphocyte marker genes CD8A, CD8B, and CD3D, the

chemokine CCL5, immune receptor KLRK1, cytolytic marker GZMK, and immunomodulatory cell surface marker SLAMF7 (all 10/10). With the exception of SLAMF7, each of these genes is used in the deconvolution algorithms' reference gene sets. The other most common DEGs not present in the reference sets include THEMIS, TIGIT, and TRBC2 (all 9/10, Supplementary Data 5), which are T cell-specific proteins. THEMIS is known to play a critical role in CD4+ CD8+ thymocyte development, TIGIT is a T-cell specific co-inhibitory molecule, and the latter consists of the constant region of the T cell receptor beta chain [24]. IPA further corroborated differential lymphocyte infiltration and activity, with the most frequent dysregulated pathways in hot clusters pertaining to leukocyte extravasation signaling and phospholipase-related signaling (Supplementary Data 6), with the most commonly activated upstream regulators as IFN-alpha, NF-kB, CD3, and TNF (Supplementary Data 7). The gene ontology of most commonly activated pathways again pertained to leukocyte and lymphocyte chemotaxis (Supplementary Data 8). Dysregulated pathways, activated upstream regulators, and gene ontology predicted by IPA on the filtered gene sets were unchanged from the unfiltered set.

For the 9 CD4+ memory T-cell infiltration phenotypes, the DEGs most commonly expressed were the T-cell receptor-CD3 complex transcript UBASH3A, cell adhesion and co-stimulatory CD2, and immunoglobulin gene IGLV2-8 (all 8/9). With the exception of IGLV2-8, the genes are defined in the deconvolution algorithm reference sets for immune content estimation. The most frequently dysregulated pathways for CD4+ memory T cell was Th2 signaling (Supplementary Data 6), with TNF as the most commonly predicted activated upstream regulator (Supplementary Data 7). For CD4+ memory T cells, the diseases and functions ontology of activated pathways was associated with chemotaxis, cell migration, and cell homing (Supplementary Data 8). IPA on filtered gene sets implicated dysregulation of the phospholipase C signaling, integrin signaling, and several immune signaling pathways in hot samples, with TNF again predicted as the activated upstream regulator, and ontologies related to cellular movement and epithelial cell differentiation.

For the 7 B-cell infiltration phenotypes, the most commonly expressed DEGs were B-cell receptor complex protein CD79A, IgG-receptor FCRL5, and IG heavy chain genes IGHD, IGHM, IGHG1, IGHG2, IGHG3, IGHG4, and IGHGP (all 7/7). The IGHD, IGHM, and CD79A transcripts are present in the reference gene sets. The most frequently dysregulated pathways for B-cell infiltration entailed opioid signaling, B cell receptor signaling, Th17 pathway activation, and NFAT in immune response regulation (Supplementary Data 6). The most common upstream regulator predicted to be activated was IL1B (Supplementary Data 7). For B cells, the diseases and functions ontology of activated pathways was associated with antigen presenting cell migration, and cell homing and movement (Supplementary Data 8). IPA on filtered gene sets implicated dysregulation of the phospholipase C signaling and dendritic cell maturation pathways in hot samples, with RELA predicted as the activated upstream regulator, and ontologies related to cellular movement.

Of the 3 composite Helper T-cell phenotypes analyzed, 60 transcripts were identified as the most commonly differentially expressed, with 36 in the reference sets.

Of the remaining 24 transcripts, 6 have identified immunomodulatory activity (TIGIT, TLR10, MIR146A, IL4I1, TIFAB, KLHDC7B), 6 encode immunglobulin proteins (IGLC7, IGHA1, IGHGP, IGLV3-9, IGLV3-21, IGLV9-49), and 3 encode immunoglobulin receptors (FCRL3, FCRLA, FCRL1). Others include proteasome-associated UBD, secretory protein FDCSP, MIAT, which is a lincRNA implicated in spliceosomal regulation, and 6 have uncharacterized function in the immune system (TBC1D27, RP11-861A13.4, RP11-693J15.5, GALNT15, OR2IP, ALPK2). The most frequently dysregulated pathway was Th2 signaling (Supplementary Data 6), with TNF, IL1B, IRF4, and CD28 as the most commonly predicted activated upstream regulators (Supplementary Data 7). For Helper T-cells, the diseases and functions ontology of activated pathways was associated with cell migration, movement, and homing (Supplementary Data 8). IPA on filtered gene sets implicated dysregulation of the neuroinflammation signaling pathway and B cell signaling pathway in lupus in hot samples, with IL1B, TNF, NF-kappaB, and IFN-gamma predicted as the activated upstream regulators, and ontologies related to cellular movement and inflammatory response.

Of the 2 NK cell phenotypes analyzed, 58 DEGs were identified as the most commonly differentially expressed, with 28 in the reference sets. Of the other 30 transcripts, 2 were NK cell specific (KLRC2, FGFBP2), 10 involved in immune signaling (IL8, CCL3, CCL4L2, XCL2, DGKK, SH2D1B, FCRL6, TRGV9, TRGV10, SERPINA3), 5 with immunomodulatory activity (LGALS9B, EOMES, GPR174, GPR114, MYOM2), 3 transcriptional regulators (MYBL1, ZNF683, ZNF80), 1 inhibitor of enzymatic degradation (TIMP4), and 9 with uncharacterized function in the immune system (LINC00299, RP11-1094M14.4, AC069363.1, RP11-1094M14.5, SOD1P3, SAMD3, UTS2, DRAXIN, AP3B2). The most commonly dysregulated pathways were those corresponding to acute phase response signaling and apoptosis signaling (Supplementary Data 6), with STAT1, TLR9, CD28, and IL18 as the most commonly predicted activated upstream regulators (Supplementary Data 7). The diseases and functions ontology of activated pathways was again associated with cell migration, movement, and homing (Supplementary Data 8). IPA on filtered gene sets implicated dysregulation of the neuroinflammation signaling and LXR/RXR metabolic pathways in hot samples, with CSF3 predicted as the activated upstream regulator, and ontologies related to cellular movement and activation of various immune cell types.

In the 15 mast cell tissue-cell pairs, the most common DEGs in hot clusters were mast cell chymase CMA1 (15/15), and IgE-receptor MS4A2, mast cell secretory enzymes CTSG and CPA3, and HDC, which is necessary for histamine synthesis (all 14/15). All of which are transcripts used in the deconvolution algorithms to estimate mast cell content. The most frequently dysregulated pathways for mast cells corresponded to THOP1 signaling pathway (Supplementary Data 6), with the most common upstream regulators IL1B and TNF (Supplementary Data 7). The gene ontology of most commonly activated pathways corresponded to chemotaxis and cell migration (Supplementary Data 8). IPA on filtered gene sets implicated dysregulation of the phospholipase C signaling, dendritic cell maturation, THOP1 in Alzheimer’s, and Wnt/Beta-catenin pathways in hot samples, with lipopolysaccharide predicted as the

activated upstream regulator, and ontologies related to cellular movement and myeloid cell response.

For the 10 monocyte phenotypes, S100A12 and S100A8 were consistently identified as upregulated (both 10/10), with the former present in the gene reference set. Both transcripts encode molecules known to induce monocyte activation and cytokine production. The most frequently dysregulated pathway for monocytes was TREM1 signaling (Supplementary Data 6), with TNF, IL1A, and TGM2 as the most commonly predicted activated upstream regulators (Supplementary Data 7). Disease and function ontologies indicated that the most commonly activated pathways in monocyte-hot samples were associated with chemotaxis, transmigration, and leukocyte and lymphocyte migration (Supplementary Data 8). IPA on filtered gene sets implicated dysregulation of the LXR/RXR metabolic pathway in hot samples, with IL1A, IL1B, IL6, TNF, and OSM predicted as the activated upstream regulators, and ontologies related to cellular movement, inflammatory response, and angiogenesis.

Of the 27 most common DEGs across the 3 neutrophil-tissue pairs, 13 were present in the reference gene sets. The remaining 14 transcripts, 3 have been identified with myeloid cell transmembrane receptors (IL1R2, CLEC4D, CACNA1E), 2 transcriptional regulators (ZDHHC1, NFE4), 2 with documented but unclear function in innate immunity (GLT1D1, KRT23), 1 immunomodulatory molecule (NLRP12), and 6 non-coding RNAs without characterized function in immunity (RP11-44F14.1, RP11-76E17.4, RP11-1220K2.2, RP11-76E17.3, LINC00211, LINC00694). The most commonly dysregulated pathway was Th2 pathway (Supplementary Data 6), with activated upstream regulator being CSF2 (Supplementary Data 7), and gene ontology associated with chemotaxis and cell movement of granulocytes and neutrophils (Supplementary Data 8). IPA on filtered gene sets implicated dysregulation of the nitric oxide production, hepatic fibrosis signaling, and LXR/RXR metabolic pathways in hot samples, with OSM predicted as the activated upstream regulator, and ontologies related to cellular movement.

Of the 2 dendritic cell phenotypes, 25 DEGs were found to be commonly expressed between hot and cold clusters, of which 8 transcripts were not in signature gene sets. One transcript (CLEC4G) corresponded to gene product participates in antigen uptake and internalization, 3 transcripts with immunomodulatory function (S100A7A, IL4I1, CRB2), 3 immunoglobulin genes (IGHA2, IGLV1-40, IGLV3-1), and 1 secretory protein (COL6A5). The most frequently dysregulated pathway corresponded to dendritic cell maturation (Supplementary Data 6), with the most common upstream regulators identified as CSF2 and IL13 (Supplementary Data 7). The gene ontology of most commonly activated pathways again pertained to chemotaxis and cell migration (Supplementary Data 8). IPA on filtered gene sets implicated dysregulation of the GP6 signaling and dendritic cell maturation pathways in hot samples, with IL1A, IL1B, IL17C/R, IL22, OSM, TNF, and IFN-gamma predicted as the activated upstream regulators, and ontologies related to cellular chemotaxis and immune response.

The most commonly expressed DEGs among the 23 aggregate myeloid infiltration phenotypes were MARCO (20/23) and CD209 (19/23), which is a cell-surface protein on both macrophage and dendritic cells. Both are transcripts in the reference

gene sets used to define content of macrophage subtypes and dendritic cell subtypes. The most frequently dysregulated pathway for myeloid infiltration was the dendritic cell maturation pathway (Supplementary Data 6), with TGM2 as the most commonly predicted activated upstream regulator (Supplementary Data 7). Disease and function ontologies again indicated that the most commonly activated pathways in myeloid-hot samples were associated with chemotaxis, transmigration, and leukocyte and lymphocyte migration (Supplementary Data 8). IPA on filtered gene sets implicated dysregulation of the dendritic cell maturation pathway in hot samples, with IL6 and TNF predicted as the activated upstream regulators, and ontologies related to cellular migration.

Of the 18 aggregate lymphoid infiltration phenotypes, the most common DEG in hot clusters were immunoglobulin proteins IGLV2-14 (16/18), and IGHG3, IGHM, and IGKV3-11 (all 15/18). Of these, only IGHM is a marker gene in the reference sets. The most frequently dysregulated pathway for lymphoid infiltration was Phospholipase C signaling (Supplementary Data 6), with TNF as the most commonly predicted activated upstream regulator (Supplementary Data 7). The gene ontology of most commonly activated pathways again pertained to leukocyte and lymphocyte chemotaxis (Supplementary Data 8). IPA on filtered gene sets implicated dysregulation of the dendritic cell maturation pathway in hot samples, with IL4 predicted as the activated upstream regulator, and ontologies related to cellular migration and chemotaxis.

Supplementary Note 7: Phenotype dropout during differential expression analysis

We observed that drop out is largely driven by our stringent requirement that an infiltration phenotype have at least 6 samples assigned to hot/cold clusters across the 3 deconvolution algorithms. Our consensus hot/cold assignment algorithm yielded 130/189 phenotypes with enough samples to proceed with DEG. Of these 130 that underwent different, 123 pass all cutoffs (at least 5 transcripts with log-fold change (logFC) >= 2 and false discovery rate significance values (FDR) < 0.01). The majority of drop outs occur during the consensus hot/cold assignment procedure. We speculate that using a larger data set would result in a much smaller fraction of phenotypes that are excluded from downstream analysis due to our sample number requirements. (Of note, 21 infiltration phenotypes featured 4 or 5 consensus hot samples and were therefore excluded from DEG). Furthermore, 7/130 phenotypes that drop out due to inability to reach DEG cutoffs have an average of 17 signature genes (reference genes in xCell and CIBERSORT) at 1.0 <= logFC <= 2.0 and FDR < 0.01. We also note that 3/7 of these infiltration phenotypes feature the lowest variance in xCell/CIBERSORT Absolute scores (bottom 5 of 189) and thus their drop out due to lacking transcripts with logFC >=2 is not unexpected. Overall, this implies that our dropout during DEG is not driven by noise, but instead a byproduct of applying strict significance thresholds.

Supplementary Note 8: Differential expression using quintiles.

We show that similar results can also be derived by using alternative approaches for assigning samples to "immune-rich" and "immune-depleted" clusters. For each

phenotype, we computed quintiles in each deconvolution output, and compared "consensus" top and bottom quintiles (sample consistently in top or bottom quintile across 3 deconvolutions for a given phenotype). The results of this differential expression analysis do not meaningfully change compared to the clustering approach. For example, in our macrophage phenotypes, the current revised analysis using consensus k-means clustering yields as top DEGs *C1QB* (18/21 tissues), *VSIG4* (17/21), *MARCO* (17/21), and C1QC and *CD163* (16/21). The consensus quintiles approach yields CD163 (23/23), C1QC, C1QB, and VSIG4 (the latter 3 all 22/23). This effect is recapitulated in the case of our CD8 T-cell phenotype as well. Our k-means approach yielded as top DEGs CD8A, CD8B, and CD3D, CCL5, and KLRK1(all 10/10). Using the consensus quintile approach yielded CD8A, CD8B (14/15), CCL5 (13/15), CD3D (12/15), KLRK1 (11/15). We found similar results across the other 10 cell types, where the major DEGs recovered through the quintiles approach to assignment are largely identical to those obtained via consensus clustering.

In this approach, 162/189 phenotypes featured at least 6 samples consistently identified in the top/bottom quintiles of each deconvolution. These samples were then considered for DEG analysis. However, only 136/162 phenotypes (84%) passed our DEG cutoffs. This is much lower percentage compared to our analysis using the consensus k-means approach, where 123/130 phenotypes (94.6%) passed our DEG cutoffs (dropout due to DEG failing to yield "significant outcome"). This implies that our k-means procedure is more capable of finding stable groupings that feature substantial transcriptomic differences at the outset relative to alternative methods. In so doing, the method also effectively filters noise for downstream analysis. For example, the choice of arbitrary, pre-determined cutoffs such as quintiles ignores patterns in the data distribution that can be observed by an automated clustering algorithm. If the distribution of macrophages in lung tissue were a mixture of Gaussians (with separate Gaussian parameter values for cold, intermediate, and hot), then a consensus clustering approach would discover the groupings best.

Overall, our method was complementary and perhaps an improved approach to another procedure for identifying "extreme" samples. It generally recovered the same transcriptomic differences as a quintile-based approach, without substantial loss of information after clustering relative to alternative approaches. Increased sample size would only improve the clustering algorithm because the differential expression analysis would not be impacted as much by the original sample drop out.

Supplementary Note 9: Overlap of hot clusters across clustering approaches

Overall, there are 9 whole blood samples that are labeled "hot" in both the k-means clustering analysis and the quintiles analysis for CD8 T cells. We show this graphically in Supplementary Figure 12. Supplementary Figure 12 displays a heatmap of "hot" cases for whole blood samples across the three approaches (k-means clustering, top quintile, top two quintiles), without clustering and aligned by sample (each column represents the same sample). All "hot" samples identified by the k-means approach are "hot" samples in the top quintile approach, but not vice versa.

Supplementary Note 10: Stability of clustering algorithm to down-sampling

We demonstrated that the hot/cold assignments are robust to subsampling by proceeding as follows. We randomly selected 6 infiltration phenotypes (Adrenal Gland – Macrophages Colon – Sigmoid – Macrophages, Colon Transverse – CD4 memory, Esophagus – Mucosa – Mast cells, Lung – Myeloid cells, and Small Intestine – B cells), and we downsampled to 50% of the total number of samples. After performing the consensus clustering procedure across the 3 deconvolution algorithms on each of the downsampled phenotypes, we found that every sample that was labeled “hot” in the subsampling was also labelled “hot” in the original assignments (with the full sample sizes). This was the same for “cold” clusters. Therefore, subsampling did not introduce any new samples into the “hot” or “cold” clusters in our simulations. This effectively demonstrates the stability of the procedure in producing consistent assignments as hot and cold.

Supplementary Note 11: Sex associations with breast tissue

Temporal dependent changes (for example, the transition from lactating states to non-lactating states and menopause status) have been associated with an altered T cell response[3], and T cells have been associated with lobule localization (with higher densities of CD8+ T cells compared to CD4+ T cells)[4]. Furthermore, female breast may harbor a higher population of antigen-presenting cells to protect against potential infections compared to male breasts, which are less exposed to infection (e.g. mastitis). To further assess the differences in the immune content of breast samples between males and females, we applied t-distributed stochastic neighbor embedding (t-SNE)[5] to the 22 immune cell scores from CIBERSORT-Absolute. The two t-SNE components displayed visual differences in clustering between males and females (Supplementary Figure 16).

Supplementary Note 12: Findings in aged blood support previous research

Compositional differences have been previously observed in aged blood, including myeloid-biased differentiation[6], a decline in the ratio of CD4 to CD8 T cells[7], and a rise in NK cells[8,9]. While our original analysis also identified an increase in NK cells (P = 0.028; not significant after FDR correction), we additionally calculated CD4:CD8 T cell and Myeloid:Lymphoid cell ratios in whole blood samples using CIBERSORT and xCell deconvolution estimates. We performed a rank-inverse normal transformation on these ratios to minimize outlier influence and analyzed these ratios using a similar regression model. Our model identified decreased CD4:CD8 T cell ratio and myeloid-skewing in aged blood (Age-CD4:CD8 T cells ratio association: Empirical Brown’s P = 0.035; Age-Myeloid:Lymphoid cell ratio association: Empirical Brown’s P = 0.024) (Supplementary Figure 17).

Supplementary Note 13: EBM *P*-values when including relative and absolute measures

The combined Empirical Brown's method *P*-values leverage signals in the compositional "immune cell" space and absolute "tissue" space for improved power. For example, in the genetic analysis, performing Empirical Brown's method on just the absolute results (not including the CIBERSORT-relative analysis) returns 21 significant phenotypes. This is less significant findings than additionally including the CIBERSORT-Relative analysis (31). Yet, we note that this is still more than each of the separate analyses (15, 16, 16).

Supplementary Note 14: Evaluating potential EBM *P*-value inflation

To test whether Empirical Brown's method (EBM) could inflate *P*-values in our study, we performed shuffled data experiments using our original finding of rs648299 and its association with the helper T cell phenotype in thyroid tissue. In the original study using real data, this SNP has an EBM *P*-value = 7.5×10^{-10}, derived from *P*-values = 5.7×10^{-7}, 3.3×10^{-9}, and 9.8×10^{-4} in CIBERSORT-Rel, CIBERSORT-Abs, and xCell separate analyses.

In our experiment, we used the covariate-adjusted phenotype values. Each individual's phenotype values are assigned to a new individual, such that individual *i* who is assigned the CIBERSORT-Rel value from individual *j* will also be assigned the CIBERSORT-Abs and xCell values from individual *j*. Sampling was performed without replacement. In this way, the covariance matrix between CIBERSORT-Rel, CIBERSORT-Abs, and xCell scores is preserved and identical to the original data (which is used in the Empirical Brown's method). We next analyzed the association between the original rs648299 and the new, shuffled phenotype for CIBERSORT-Rel, CIBERSORT-Abs, and xCell using a simple linear model. The *P*-values from the three linear models were combined using EBM. This process was repeated for 10,000 simulations, and *P*-value inflation was assessed by analyzing false positive rate. False positive rate was calculated by identifying the percentage of simulations where $P < 0.05$.

Our results found a false positive rate of 0.0529, 0.0504, and 0.0483 for CIBERSORT-Rel, CIBERSORT-Abs, and xCell separate analyses. Using the combined EBM framework, the false positive rate was lower, 0.0388. We also note that there was a single simulation where CIBERSORT-Absolute analysis returned a *P*-value of 1.9×10^{-6}, which is lower than a Bonferroni-corrected *P*-value threshold of $P < 0.05/10000 = 5.0 \times 10^{-6}$. In this particular case with the strong CIBERSORT-Absolute association, analysis in CIBERSORT-Relative returned $P = 7.3 \times 10^{-3}$ and analysis in xCell returned $P = 4.4 \times 10^{-2}$. The combined EBM *P*-value was 3.6×10^{-5}, which is greater than the $P < 5.0 \times 10^{-6}$ Bonferroni cut-off. Thus, the combined *P*-value framework helped decrease the significance of this *P*-value such that it would not be rejected under a Bonferroni correction by leveraging the lower strength of association within the other deconvolution method analyses.

Overall, in this shuffled data experiment, there did not appear to be *P*-value inflation.

Supplementary Note 15: Using an aggregate expression analysis versus deconvolution

It can be argued that the significant findings would be discovered with a more standard analysis, without the need for deconvolution. We analyzed the association between rs6482199 and the Helper T cell phenotype in thyroid tissue as a representative example (and a positive result). We extracted the signature genes for the helper T cells used by CIBERSORT, calculated the median expression of these genes in the thyroid samples, and tested for the association between rs6482199 and the aggregate value. Here, we found that rs6482199 was associated with the aggregated median expression level of the helper T cell phenotype at a less significant *P*-value ($P = 0.0058$). We repeated this type of analysis using our second most significant association, rs56234965 with lymphocytes in sigmoid colon tissue. We identified all signature genes from all cell types in the lymphocyte phenotype, and found that the variant also correlated with the new aggregate phenotype, although less significantly so ($P = 0.00059$). These results suggest that greater resolution is obtained through deconvolution compared to a more ad hoc or simpler procedure for cell type inference, although similar results can be derived using either method.

We repeated this analysis in context of the hot vs cold differential expression analysis. For a particular cell type category, we identified the signature genes used for deconvolution of that cell type. Then, for each sample, we aggregated the expression levels of the gene set by calculating the median value. This aggregated median expression level was used as a surrogate for the amount of the immune cell type in the sample. We next correlated each gene in the transcriptome against the aggregate measure. We compared the significantly correlated genes from this analysis from those that were discovered by performing a differential expression analysis of clustered “hot” and “cold” samples of the respective deconvolution scores.

By performing this analysis for macrophages within 10 different tissues, we found that the majority of highly significant, highly correlated genes corresponded to DEGs discovered in our differentially expression analysis (genes with $0.8 <$ correlation < 0.95 and $10^{-80} <$ *P*-values $< 10^{-35}$ often corresponded to DEGs with logFC >= 2 and FDR $< 10^{-20}$). Furthermore, this approach yields our most common DEGs discussed in the manuscript as highly significant, highly correlated genes as well (e.g. MARCO, CD163, VSIG4, C1QB featured $0.6 <$ correlation < 0.95 in many tissues, with *P*-values $< 10^{-5}$).

Overall, we believe that defining simpler metrics, such as aggregate signature gene expression levels, is a more straightforward, alternative approach to quantifying immune content in bulk samples. Previous literature has used similar methods for estimating immune content in bulk tumor sequencing[10]. However, deconvolution is preferable to the use of gene clusters or metagenes by weighting different genes separately and combining them non-linearly. As such, we chose to use both CIBERSORT and xCell to most accurately estimate the amounts of immune cells. The two methods are reliant on vastly different core algorithms and references for immune content estimation. Therefore, each algorithm has its own implicit biases but its own distinct strengths. In particular, CIBERSORT performs well with respect to

measurement error and amount of unknown sample content (which describe parts of the mixture, such as tumor content, not accounted for in the signature matrix)[11]. Additionally, CIBERSORT has shown accurate estimation of closely related cell types by accounting for multicollinearity[12] and returns *P*-values that test whether any of the reference cell types were present in the input sample. On the other hand, xCell is particularly robust to any batch effects by using a ssGSEA framework[12,13]. This is especially important in our dataset given the multi-institutional scale of the GTEx project and potential risk of technical artifacts. In addition, xCell has the highest true negative rate relative to other algorithms in simulations[12] (e.g. % of time that the deconvolution algorithm will create a score of 0 or a null score for a cell type not actually present in the sample). Therefore, leveraging both xCell and CIBERSORT was optimal to identify statistical signals.

In summary, we elected to use more sophisticated methods because of their desirable modelling properties that have shown superior performance in previous studies. We show that both the deconvolution approaches and the aggregate expression level converge on similar results. Additionally, using deconvolution can lead to more accurate and more precise results, especially in conjunction with our requirement of agreement in the results across each method.

Supplementary Note 16: Two-sided iQTL/eQTL enrichment test

While a one-sided test focuses on the enrichment of iQTL/eQTL overlap, it may not be fair to rule out depletion of this overlap in some phenotypes. We also performed a two-sided test, and found that some phenotypes do have an under-representation of iQTL/eQTL overlap compared to random segregation. However, the eQTL-enrichment of results was more significant than eQTL-underrepresentation: the grouped -log10 *P*-values of eQTL-enriched phenotypes versus eQTL-depleted were significantly different (Mann-Whitney U test, $P = 2.77 \times 10^{-9}$).

Supplementary References

1. Moldoveanu, B. *et al.* Inflammatory mechanisms in the lung. *J Inflamm Res* **2**, 1-11 (2009).
2. Kirby, J.A., Bone, M., Robertson, H., Hudson, M. & Jones, D.E. The number of intraepithelial T cells decreases from ascending colon to rectum. *J Clin Pathol* **56**, 158 (2003).
3. Betts, C.B. *et al.* Mucosal Immunity in the Female Murine Mammary Gland. *J Immunol* **201**, 734-746 (2018).
4. Degnim, A.C. *et al.* Immune cell quantitation in normal breast tissue lobules with and without lobulitis. *Breast Cancer Res Treat* **144**, 539-49 (2014).
5. Maaten, L.v.d. & Hinton, G. Visualizing data using t-SNE. *Journal of machine learning research* **9**, 2579-2605 (2008).
6. Pang, W.W. *et al.* Human bone marrow hematopoietic stem cells are increased in frequency and myeloid-biased with age. *Proceedings of the National Academy of Sciences* **108**, 20012-20017 (2011).
7. Amadori, A. *et al.* Genetic control of the CD4/CD8 T-cell ratio in humans. *Nature medicine* **1**, 1279 (1995).
8. Valiathan, R., Ashman, M. & Asthana, D. Effects of ageing on the immune system: infants to elderly. *Scandinavian journal of immunology* **83**, 255-266 (2016).
9. Facchini, A. *et al.* Increased number of circulating Leu 11+(CD 16) large granular lymphocytes and decreased NK activity during human ageing. *Clinical and experimental immunology* **68**, 340 (1987).
10. Bindea, G. *et al.* Spatiotemporal dynamics of intratumoral immune cells reveal the immune landscape in human cancer. *Immunity* **39**, 782-795 (2013).
11. Monaco, G. *et al.* RNA-Seq signatures normalized by mRNA abundance allow absolute deconvolution of human immune cell types. *Cell reports* **26**, 1627-1640. e7 (2019).
12. Sturm, G. *et al.* Comprehensive evaluation of transcriptome-based cell-type quantification methods for immuno-oncology. *Bioinformatics* **35**, i436-i445 (2019).
13. Aran, D., Hu, Z. & Butte, A.J. xCell: digitally portraying the tissue cellular heterogeneity landscape. *Genome biology* **18**, 220 (2017).
