# Supplementary material for: Demographic and genetic factors influence the abundance of infiltrating immune cells in human tissues
Source: Nat Commun. 2020 May 5;11:2213. doi: 10.1038/s41467-020-16097-9 (PMC7200670; doi:10.1038/s41467-020-16097-9)
Supplement: Supplementary file 3 — Reporting Summary [file 41467_2020_16097_MOESM3_ESM.pdf]

nature research

Corresponding author(s): *Double-blind peer review submissions: write DBPR and your manuscript number here instead of author names.*

Last updated by author(s): *YYYY-MM-DD*

# Reporting Summary

Nature Research wishes to improve the reproducibility of the work that we publish. This form provides structure for consistency and transparency in reporting. For further information on Nature Research policies, see Authors & Referees and the Editorial Policy Checklist.

## Statistics

For all statistical analyses, confirm that the following items are present in the figure legend, table legend, main text, or Methods section.

| n/a | Confirmed | |
|---|---|---|
| ☐ | ☒ | The exact sample size (*n*) for each experimental group/condition, given as a discrete number and unit of measurement |
| ☐ | ☒ | A statement on whether measurements were taken from distinct samples or whether the same sample was measured repeatedly |
| ☐ | ☒ | The statistical test(s) used AND whether they are one- or two-sided<br>*Only common tests should be described solely by name; describe more complex techniques in the Methods section.* |
| ☐ | ☒ | A description of all covariates tested |
| ☐ | ☒ | A description of any assumptions or corrections, such as tests of normality and adjustment for multiple comparisons |
| ☐ | ☒ | A full description of the statistical parameters including central tendency (e.g. means) or other basic estimates (e.g. regression coefficient) AND variation (e.g. standard deviation) or associated estimates of uncertainty (e.g. confidence intervals) |
| ☐ | ☒ | For null hypothesis testing, the test statistic (e.g. *F*, *t*, *r*) with confidence intervals, effect sizes, degrees of freedom and *P* value noted<br>*Give P values as exact values whenever suitable.* |
| ☐ | ☒ | For Bayesian analysis, information on the choice of priors and Markov chain Monte Carlo settings |
| ☐ | ☒ | For hierarchical and complex designs, identification of the appropriate level for tests and full reporting of outcomes |
| ☐ | ☒ | Estimates of effect sizes (e.g. Cohen's *d*, Pearson's *r*), indicating how they were calculated |

*Our web collection on statistics for biologists contains articles on many of the points above.*

## Software and code

Policy information about availability of computer code

| | |
|---|---|
| Data collection | Data collection The following software was used: STAR, GeneMania, samtools, Ingenuity Pathway Analysis. R software packages were used: phenoscanner, ggplot2, data.table, cowplot, EmpiricalBrownsMethod, pheatmap, limma, tsne, stringr, DESeq2, edgeR, reshape2, parallel, e1071 |
| Data analysis | All computer code used to support the findings in the manuscript are located at the following GitHub repository: https://github.com/drewmard/GTEx_infil |

For manuscripts utilizing custom algorithms or software that are central to the research but not yet described in published literature, software must be made available to editors/reviewers. We strongly encourage code deposition in a community repository (e.g. GitHub). See the Nature Research guidelines for submitting code & software for further information.

## Data

Policy information about availability of data

All manuscripts must include a data availability statement. This statement should provide the following information, where applicable:

- Accession codes, unique identifiers, or web links for publicly available datasets
- A list of figures that have associated raw data
- A description of any restrictions on data availability

The genetic data that supports the findings of this study can be found under dbGaP study accession phs000424.v8.p2 as the v6 release. The gene expression information can be found using the v7 release from gtexportal.org.

# Field-specific reporting

Please select the one below that is the best fit for your research. If you are not sure, read the appropriate sections before making your selection.

☒ Life sciences ☐ Behavioural & social sciences ☐ Ecological, evolutionary & environmental sciences

For a reference copy of the document with all sections, see nature.com/documents/nr-reporting-summary-flat.pdf

# Life sciences study design

All studies must disclose on these points even when the disclosure is negative.

| | |
|---|---|
| Sample size | The v6 GTEx release was used for genetic data and the v7 GTEx release was used for expression data. The v7 release contains 11,688 samples, spanning 53 different tissues/sample types from 714 donors. The v6 release has 450 total individuals. The sample size varies across tissues. Individuals do not contribute samples for every tissue type. The sample sizes used in analysis are the maximum sample possible with the available data. This sample size is enough to detect large effect sizes, but would miss most minor and smaller effects. |
| Data exclusions | No data was excluded. |
| Replication | To replicate GWAS associations with infiltration phenotypes, we used previous GWAS in larger cohorts (e.g. UK Biobank). Genetic variants associated with an infiltration phenotype were also tested to see whether they were associated with any related phenotypes in separate, larger studies. We also observed an enrichment of significant genetic associations that have been identified as genome-wide significant in previous GWAS. |
| Randomization | Any relevant covariate information that could confound findings were used as covariates in analysis: gene expression based principal components, age, sex, genotype array, death classification. It is assumed that many potential confounders are appropriately accounted for in analysis. However, neither randomization nor any splitting of individuals into different groups were performed. |
| Blinding | Blinding for researchers was not necessary: we used an unbiased approach to identify new associations. Furthermore, blinding for donor research participants was not relevant to downstream research. |

# Reporting for specific materials, systems and methods

We require information from authors about some types of materials, experimental systems and methods used in many studies. Here, indicate whether each material, system or method listed is relevant to your study. If you are not sure if a list item applies to your research, read the appropriate section before selecting a response.

## Materials & experimental systems

| n/a | Involved in the study | |
|---|---|---|
| ☒ | ☐ | Antibodies |
| ☒ | ☐ | Eukaryotic cell lines |
| ☒ | ☐ | Palaeontology |
| ☒ | ☐ | Animals and other organisms |
| ☐ | ☒ | Human research participants |
| ☒ | ☐ | Clinical data |

## Methods

| n/a | Involved in the study | |
|---|---|---|
| ☒ | ☐ | ChIP-seq |
| ☒ | ☐ | Flow cytometry |
| ☒ | ☐ | MRI-based neuroimaging |

## Human research participants

Policy information about studies involving human research participants

| | |
|---|---|
| Population characteristics | The v6 GTEx release was used for genetic data and the v7 GTEx release was used for expression data. The v7 release contains 11,688 samples, spanning 53 different tissues/sample types from 714 donors. The v6 release has 450 total individuals. The sample size varies across tissues. Individuals do not contribute samples for every tissue type. >80% individuals are European. Age ranges from 20 to 79 and is >60% male. Individuals contributed a mode of 19 tissues. |
| Recruitment | GTEx participants were deceased. Participants/participants' families chose to donate tissue and organ samples to GTEx project. |
| Ethics oversight | GTEx consortium. |

Note that full information on the approval of the study protocol must also be provided in the manuscript.
