# Supplementary material for: Demographic and genetic factors influence the abundance of infiltrating immune cells in human tissues
Source: Nat Commun. 2020 May 5;11:2213. doi: 10.1038/s41467-020-16097-9 (PMC7200670; doi:10.1038/s41467-020-16097-9)
Supplement: Supplementary file 4 — Description of Additional Supplementary Files [file 41467_2020_16097_MOESM4_ESM.pdf]

Description of Additional Supplementary Files

Supplementary Data 1: List of infiltration phenotypes after filtering and kept for downstream analysis.

Supplementary Data 2: The 189 post-filtered infiltration phenotypes from CIBERSORT-Absolute were tested for association with the first four gene expression-based principal components using a linear model. All *P*-values (189 phenotypes * 4 PCs = 756 total tests) were adjusted using a FDR correction. The minimum *P*-value for each infiltration phenotype (from 4 total *P*-values) is displayed, along with the adjusted value.

Supplementary Data 3: Assignment of samples from infiltration phenotypes to hot and cold phenotypes, and covariate data used in differential expression. Each sample is labeled “hot” or “cold” based on consistent classification across the 3 deconvolution methods.

Supplementary Data 4: Covariates included in design matrix for differential expression for infiltration phenotypes, with “1” entailing inclusion of covariate, and “0” corresponding to exclusion. Covariate inclusion was based on sufficient representation of each level of the covariate in hot and cold clusters.

Supplementary Data 5: Differential gene expression results from infiltration phenotypes expressing at least 5 transcripts at cutoffs of log fold change > 2 and adjusted *P*-value < 0.01.

Supplementary Data 6: IPA canonical pathway analysis for hot and cold clusters across infiltration phenotypes.

Supplementary Data 7: IPA upstream regulator analysis for the hot and cold clusters across infiltration phenotypes.

Supplementary Data 8: IPA gene ontology analysis for the hot and cold clusters across infiltration phenotypes. Annotations of disease or functional association and corresponding dysregulated molecules are represented.

Supplementary Data 9: Summary of association testing between infiltration phenotypes and age or sex.

Supplementary Data 10: Genome-wide significant iQTL results of GWAS across 189 infiltration phenotypes. Shown is SNPs with the minimum *P*-values in each of the 31 infiltration phenotypes with genome-wide significant associations ($P < 5 \times 10^{-8}$) from any analysis: Empirical Brown's method, xCell only, CIBERSORT-Absolute only, or CIBERSORT- Relative only.

Supplementary Data 11: All suggested significant iQTL results from GWAS across 189 infiltration phenotypes ($P < 10^{-5}$).

Supplementary Data 12: All suggested significant ieQTLs results from GWAS across 189 infiltration phenotypes (iQTL $P < 10^{-5}$; eQTL significance from GTEx v7 Consortium analysis). This is also the list of ieQTLs used to generate the input query of ieGenes for GeneMania.

Supplementary Data 13: Gene ontology (GO) functional enrichment from large GeneMania network of ieGenes (from ieQTLs associated with infiltration phenotype at a relaxed $P < 10^{-5}$ threshold).

Supplementary Data 14: SNPs associated with multiple infiltration phenotypes at the relaxed suggestive significance threshold ($P < 10^{-5}$).

Supplementary Data 15: GeneMania-produced report describing output from the input query of associated ieGenes.

Supplementary Data 16: List of synthetic mixes simulated for testing deconvolution methods.
