# Supplementary material for: Demographic and genetic factors influence the abundance of infiltrating immune cells in human tissues
Source: Nat Commun. 2020 May 5;11:2213. doi: 10.1038/s41467-020-16097-9 (PMC7200670; doi:10.1038/s41467-020-16097-9)
Supplement: Supplementary file 19 — Supplementary Data 15 [file 41467_2020_16097_MOESM19_ESM.pdf]

GeneMANIA report

Created on : 20 August 2019 11:29:16
Last database update : 13 March 2017 00:00:00
Application version : 3.6.0

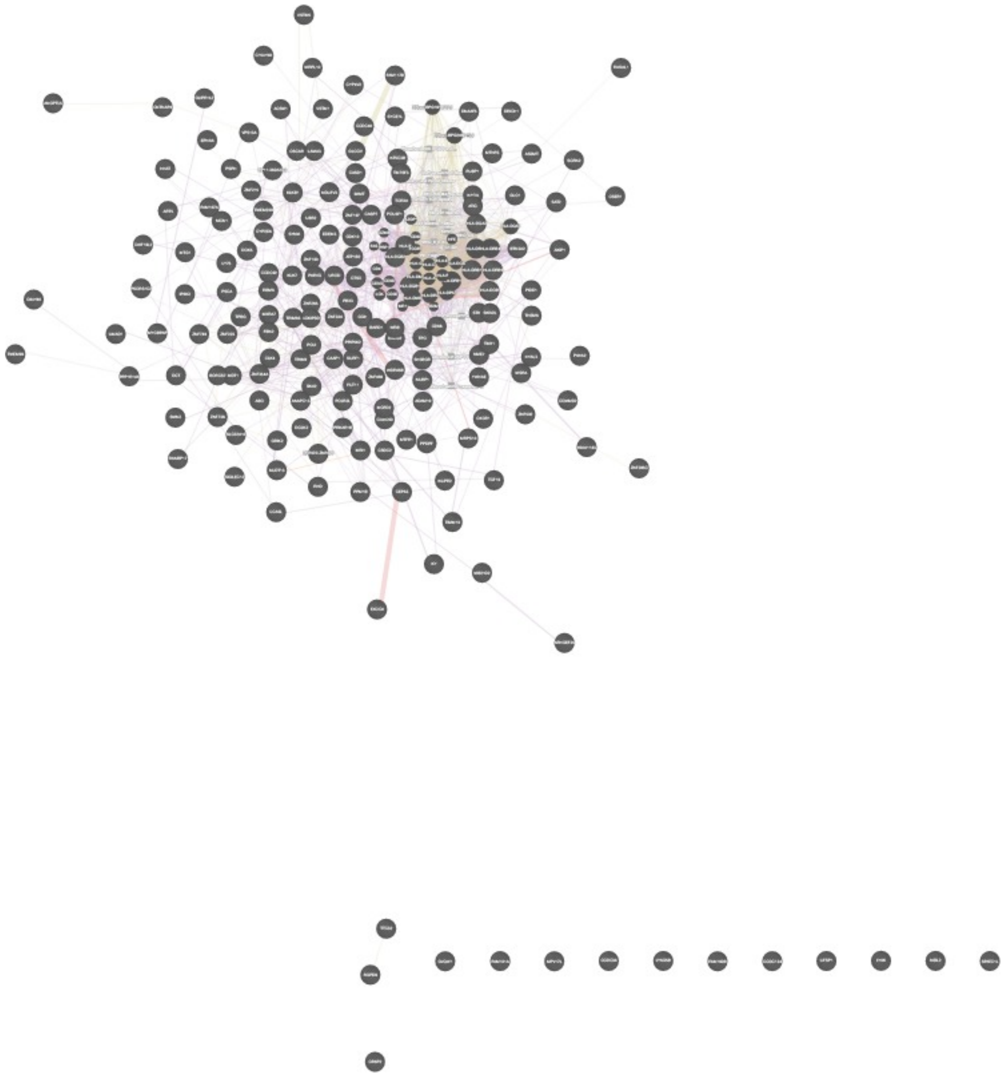

Networks

- Consolidated-Pathways-2013
- InterPro
- Co-expression
- Physical Interactions
- Shared protein domains
- Predicted

Functions

N/A

Search parameters

Organism Homo sapiens (human)

Genes ENSG00000004534 , ENSG00000006042 , ENSG00000024048 , ENSG00000037757 , ENSG00000048544 , ENSG00000053747 , ENSG00000054219 , ENSG00000054392 , ENSG00000064115 , ENSG00000066855 , ENSG00000068745 , ENSG00000072682 , ENSG00000080166 , ENSG00000080224 , ENSG00000100147 , ENSG00000100197 , ENSG00000100505 , ENSG00000101134 , ENSG00000101751 , ENSG00000103274 , ENSG00000106415 , ENSG00000108953 , ENSG00000111344 , ENSG00000113240 , ENSG00000114744 , ENSG00000115705 , ENSG00000116406 , ENSG00000117593 , ENSG00000119242 , ENSG00000120159 , ENSG00000124767 , ENSG00000125534 , ENSG00000126088 , ENSG00000127995 , ENSG00000129055 , ENSG00000129226 , ENSG00000129244 , ENSG00000130193 , ENSG00000130413 , ENSG00000131558 , ENSG00000131845 , ENSG00000132305 , ENSG00000132823 , ENSG00000134809 , ENSG00000135074 , ENSG00000135747 , ENSG00000136371 , ENSG00000136449 , ENSG00000137310 , ENSG00000137563 , ENSG00000138032 , ENSG00000138363 , ENSG00000138376 , ENSG00000138439 , ENSG00000138829 , ENSG00000138964 , ENSG00000139921 , ENSG00000141295 , ENSG00000141504 , ENSG00000141519 , ENSG00000141580 , ENSG00000141698 , ENSG00000143156 , ENSG00000145029 , ENSG00000145476 , ENSG00000145725 , ENSG00000146242 , ENSG00000146733 , ENSG00000146842 , ENSG00000148290 , ENSG00000148824 , ENSG00000149054 , ENSG00000152404 , ENSG00000152492 , ENSG00000152910 , ENSG00000154319 , ENSG00000154328 , ENSG00000156968 , ENSG00000157578 , ENSG00000159111 , ENSG00000160194 , ENSG00000160284 , ENSG00000160886 , ENSG00000162613 , ENSG00000163807 , ENSG00000164344 , ENSG00000164418 , ENSG00000164818 , ENSG00000165621 , ENSG00000165806 , ENSG00000166275 , ENSG00000166321 , ENSG00000166436 , ENSG00000166452 , ENSG00000166503 , ENSG00000166743 , ENSG00000167081 , ENSG00000167311 , ENSG00000167641 , ENSG00000167653 , ENSG00000169035 , ENSG00000169085 , ENSG00000169131 , ENSG00000169609 , ENSG00000169629 , ENSG00000170390 , ENSG00000170909 , ENSG00000172346 , ENSG00000172687 , ENSG00000173421 , ENSG00000174611 , ENSG00000175164 , ENSG00000175170 , ENSG00000175806 , ENSG00000176125 , ENSG00000177595 , ENSG00000177700 , ENSG00000178386 , ENSG00000178882 , ENSG00000179344 , ENSG00000180398 , ENSG00000180481 , ENSG00000180611 , ENSG00000182093 , ENSG00000182253 , ENSG00000182534 , ENSG00000182923 , ENSG00000183172 , ENSG00000183303 , ENSG00000183891 , ENSG00000184999 , ENSG00000185290 , ENSG00000185324 , ENSG00000185437 , ENSG00000186230 , ENSG00000186283 , ENSG00000186448 , ENSG00000186470 , ENSG00000186792 , ENSG00000187010 , ENSG00000187151 , ENSG00000187595 , ENSG00000188191 , ENSG00000189068 , ENSG00000189269 , ENSG00000196126 , ENSG00000232629 , ENSG00000196653 , ENSG00000196968 , ENSG00000197020 , ENSG00000197969 , ENSG00000198502 , ENSG00000198951 , ENSG00000204351 , ENSG00000204531 , ENSG00000204538 , ENSG00000204604 , ENSG00000204764 , ENSG00000205078 , ENSG00000205302 ,

ENSG00000205571 , ENSG00000237541 , ENSG00000234745 , ENSG00000213214 , ENSG00000213672 , ENSG00000214376 , ENSG00000214435 , ENSG00000219545 , ENSG00000240053 , ENSG00000241106 , ENSG00000241043 , ENSG00000254521 , ENSG00000256043 , ENSG00000267508

Network weighting Automatically selected weighting method

Networks

A

Abu-Odeh-Aqeilan-2014 , Agrawal-Sedivy-2010 , Aichem-Groettrup-2012 , Albers-Koegl-2005 , Alexandru-Deshaies-2008 , Alizadeh-Staudt-2000 , Andresen-Flores-Morales-2014 , Arbuckle-Grant-2010 , Arroyo-Aloy-2014 , Arroyo-Aloy-2015

B

Bahr-Bowler-2013 , Bailey-Hieter-2015 , Bandyopadhyay-Ideker-2010 , Bantscheff-Drewes-2011 , Barr-Knapp-2009 , Barrios-Rodiles-Wrana-2005 , Behrends-Harper-2010 , Behzadnia-Lührmann-2007 , Bennett-Harper-2010 , Benzinger-Hermeking-2005 , Berggård-James-2006 , Bett-Hay-2013 , Bhatnagar-Attie-2014 , Bild-Nevins-2006 B , BIOGRID-SMALL-SCALE-STUDIES , BIOGRID-SMALL-SCALE-STUDIES , Blandin-Richard-2013 , Blomen-Brummelkamp-2015 , Blomen-Brummelkamp-2015 , Bogachek-Weigel-2014 , Boldrick-Relman-2002 , Bonacci-Soubeyran-2014 , Bouwmeester-Superti-Furga-2004 , Brajenovic-Drewes-2004 , Brehme-Superti-Furga-2009 , Bruderer-Hay-2011 , Burington-Shaughnessy-2008 , Butland-Hayden-2014 , Byron-Humphries-2012

C

Cai-Conaway-2007 , Camargo-Brandon-2007 , Campos-Reinberg-2015 , Cao-Chinnaiyan-2014 , Carmon-Liu-2014 , CELL_MAP , Chen-Brown-2002 , Chen-Ge-2013 , Chen-Huang-2014 , Chen-Zhang-2013 , Christianson-Kopito-2011 , Cloutier-Coulombe-2013 , Colland-Gauthier-2004 , Consolidated-Pathways-2013 , Corominas-Iakoucheva-2014 , Couzens-Gingras-2013 , Cox-Rizzino-2013 , Coyaud-Raught-2015

D

Danielsen-Nielsen-2011 , Dart-Wells-2015 , de Hoog-Mann-2004 , Diner-Cristea-2015 , Dobbin-Giordano-2005 , Drissi-Boisvert-2015 , Drug-interactions-2013 , Dyer-Sobral-2010

E

Emanuele-Elledge-2011 , Emdal-Olsen-2015 , Ewing-Figeys-2007

F

Fenner-Prehn-2010 , Floyd-Pagliarini-2016 , Foerster-Ritter-2013 , Fogeron-Lange-2013 , Foster-Marshall-2013 , Freibaum-Taylor-2010

G

Gabriel-Baumgrass-2016 , Galligan-Howley-2015 , Gao-Reinberg-2012 , Gautier-Hall-2009 , Giannone-Liu-2010 , Glatter-Gstaiger-2009 , Gloeckner-Ueffing-2007 , Goehler-Wanker-2004 , Golebiowski-Hay-2009 , Goudreault-Gingras-2009 , Grant-

G

2010 , Greco-Cristea-2011 , Grossmann-Stelzl-2015 , Guarani-Harper-2014 , Gupta-Pelletier-2015

H

Hanson-Clayton-2014 , Hauri-Gstaiger-2013 , Havrylov-Redowicz-2009 , Havugimana-Emili-2012 , Hayes-Urbé-2012 , Hegele-Stelzl-2012 A , Hegele-Stelzl-2012 B , Hein-Mann-2015 , Hill-Livingston-2014 , HUMANCYC , Humphries-Humphries-2009 , Hutchins-Peters-2010 , Huttlin-Gygi-2015

I

I2D-BIND-Fly2Human , I2D-BIND-Mouse2Human , I2D-BIND-Rat2Human , I2D-BIND-Worm2Human , I2D-BIND-Yeast2Human , I2D-BioGRID-Fly2Human , I2D-BioGRID-Mouse2Human , I2D-BioGRID-Rat2Human , I2D-BioGRID-Worm2Human , I2D-BioGRID-Yeast2Human , I2D-Chen-Pawson-2009-PiwiScreen-Mouse2Human , I2D-Formstecher-Daviet-2005-Embryo-Fly2Human , I2D-Giot-Rothbert-2003-Low-Fly2Human , I2D-INNATEDB-Mouse2Human , I2D-IntAct-Fly2Human , I2D-IntAct-Mouse2Human , I2D-IntAct-Rat2Human , I2D-IntAct-Worm2Human , I2D-IntAct-Yeast2Human , I2D-Krogan-Greenblatt-2006-Core-Yeast2Human , I2D-Krogan-Greenblatt-2006-NonCore-Yeast2Human , I2D-Li-Vidal-2004-CORE-1-Worm2Human , I2D-Li-Vidal-2004-non-core-Worm2Human , I2D-Manual-Mouse2Human , I2D-Manual-Rat2Human , I2D-MGI-Mouse2Human , I2D-MINT-Fly2Human , I2D-MINT-Mouse2Human , I2D-MINT-Rat2Human , I2D-MINT-Worm2Human , I2D-MINT-Yeast2Human , I2D-Ptacek-Snyder-2005-Yeast2Human , I2D-Tarassov-PCA-Yeast2Human , I2D-Tewari-Vidal-2004-TGFb-Worm2Human , I2D-vonMering-Bork-2002-High-Yeast2Human , I2D-vonMering-Bork-2002-Low-Yeast2Human , I2D-vonMering-Bork-2002-Medium-Yeast2Human , I2D-Wang-Orkin-2006-EScmplx-Mouse2Human , I2D-Wang-Orkin-2006-EScmplxlow-Mouse2Human , I2D-Yu-Vidal-2008-GoldStd-Yeast2Human , IMID , Ingham-Pawson-2005 , Innocenti-Brown-2011 , INTERPRO , InterPro , IREF-BIND , IREF-BIOGRID , IREF-DIP , IREF-HPRD , IREF-INTACT , IREF-MATRIXDB , IREF-MPPI , IREF-PUBMED , IREF-SMALL-SCALE-STUDIES , IREF-SMALL-SCALE-STUDIES

J

Jeronimo-Coulombe-2007 , Jin-Pawson-2004 , Johnson-Kerner-Wichterle-2015 , Johnson-Shoemaker-2003 , Jones-MacBeath-2006 , Joshi-Cristea-2013 , Jäger-Krogan-2011

K

Kahle-Zoghbi-2011 , Kaltenbach-Hughes-2007 , Katsogiannou-Rocchi-2014 , Kim-Gygi-2011 , Kim-Major-2015 , Kneissl-Grummt-2003 , Koch-Hermeking-2007 , Kotlyar-Jurisica-2015 , Kristensen-Foster-2012 , Kärblane-Sarmiento-2015 , Kırlı-Görlich-2015

L

L

Lambert-Gingras-2015 , Lamoliatte-Thibault-2014 , Lau-Ronai-2012 , Lee-Songyang-2011 , Lehner-Sanderson-2004 A , Lehner-Sanderson-2004 B , Leng-Wang-2014 , Leung-Jones-2014 , Li-Chen-2015 , Li-Dorf-2011 A , Li-Dorf-2011 B , Li-Dorf-2014 , Li-Haura-2013 , Lim-Zoghbi-2006 , Lin-Smith-2010 , Lipp-Guthrie-2015 , Liu-Wang-2012 , Llères-Lamond-2010 , Loch-Strickler-2012 , Low-Heck-2014 , Lu-Zhang-2013 , Luo-Elledge-2009

M

Mak-Moffat-2010 , Mallon-McKay-2013 , Malovannaya-Qin-2010 , Markson-Sanderson-2009 , Maréchal-Zou-2014 , Matsumoto-Nakayama-2005 , McCracken-Blencowe-2005 , McFarland-Nussbaum-2008 , Meek-Piwnica-Worms-2004 , Milev-Mouland-2012 , miRNA-target-predictions-2013 , Miyamoto-Sato-Yanagawa-2010 , Murakawa-Landthaler-2015

N

Nakayama-Ohara-2002 , Nakayasu-Adkins-2013 , Napolitano-Meroni-2011 , Narayan-Bennett-2012 , Nathan-Goldberg-2013 , NCI_NATURE , Neganova-Lako-2011 , Newman-Keating-2003 , Nicholson-Hupp-2014 , Noble-Diehl-2008

O

Oliviero-Cagney-2015 , Olma-Pintard-2009 , Oláh-Ovádi-2011 , Oshikawa-Nakayama-2012 , Ouyang-Gill-2009

P

Panigrahi-Pati-2012 , Papp-Lamia-2015 , Perez-Hernandez-Yáñez-Mó-2013 , Perou-Botstein-1999 , Perou-Botstein-2000 , Persaud-Rotin-2009 , Petschnigg-Stagljar-2014 , PFAM , Phillips-Corn-2013 , Pichlmair-Superti-Furga-2011 , Pichlmair-Superti-Furga-2012 , Pilot-Storck-Goillot-2010 , Povlsen-Choudhary-2012

R

Ramachandran-LaBaer-2004 , Raman-Harper-2015 , Ramaswamy-Golub-2001 , Ravasi-Hayashizaki-2010 , REACTOME , Reinke-Keating-2013 , Reyniers-Taymans-2014 , Richter-Chrzanowska-Lightowlers-2010 , Rieger-Chu-2004 , Rolland-Vidal-2014 , Rosenwald-Staudt-2001 , Roth-Zlotnik-2006 , Roux-Burke-2012 , Rowbotham-Mermoud-2011 , Roy-Pardo-2014 , Roy-Parent-2013 , Rual-Vidal-2005 A , Rual-Vidal-2005 B

S

Sang-Jackson-2011 , Sato-Conaway-2004 , Schadt-Shoemaker-2004 , Scholz-Taylor-2016 , Singh-Moore-2012 , Smirnov-Cheung-2009 , So-Colwill-2015 , Soler-López-Aloy-2011 , Sowa-Harper-2009 , Stehling-Lill-2012 , Stehling-Lill-2013 , Stelzl-Wanker-2005 , Stes-Gevaert-2014 , Stuart-Kim-2003 , Suter-Wanker-2013

T

Taipale-Lindquist-2012 , Taipale-Lindquist-2014 , Takahashi-Conaway-2011 , Tarallo-Weisz-2011 , Tatham-Hay-2011 , Teixeira-Gomes-2010 , Thalappilly-Dusetti-

T

2008 , Thompson-Luchansky-2014 , Tong-Moran-2014 , Toyoshima-Grandori-2012 , Transcriptional-factor-targets-2013 , Tsai-Cristea-2012

U

Udeshi-Carr-2012

V

van Wijk-Timmers-2009 , Vandamme-Angrand-2011 , Varjosalo-Gstaiger-2013 , Varjosalo-Superti-Furga-2013 , Venkatesan-Vidal-2009 , Vermeulen-Mann-2010 , Vinayagam-Wanker-2011 , Virok-Fülöp-2011 , Vizeacoumar-Moffat-2013

W

Wagner-Choudhary-2011 , Wallach-Kramer-2013 , Wan-Emili-2015 , Wang-Balch-2006 , Wang-Cheung-2015 , Wang-He-2008 , Wang-Maris-2006 , Wang-Xu-2015 , Wang-Yang-2011 , Weimann-Stelzl-2013 A , Weimann-Stelzl-2013 B , Weinmann-Meister-2009 , Wen-Wu-2014 , Whisenant-Salomon-2015 , Wilker-Yaffe-2007 , Willingham-Muchowski-2003 , Witt-Labeit-2008 , Wong-O'Bryan-2012 , Woods-Monteiro-2012 , Woodsmith-Sanderson-2012 , Wu-Garvey-2007 , Wu-Li-2007 , Wu-Ma-2012 , Wu-Stein-2010 , Wu-Stein-2010

X

Xiao-Lefkowitz-2007 , Xie-Cong-2013 , Xie-Green-2012 , Xu-Ye-2012

Y

Yang-Chen-2010 , Yatim-Benkirane-2012 , Yu-Chow-2013 , Yu-Vidal-2011

Z

Zanon-Pichler-2013 , Zhang-Shang-2006 , Zhang-Zou-2011 , Zhao-Krug-2005 , Zhao-Yang-2011 , Zhou-Conrads-2004 , Zhou-Hanemann-2016

Genes

Gene	Description	Rank
GVQW1	GVQW motif containing 1 [Source:HGNC Symbol;Acc:HGNC:31424]	N/A
ZNF738	zinc finger protein 738 [Source:HGNC Symbol;Acc:HGNC:32469]	N/A
ZNF670-ZNF695	ZNF670-ZNF695 readthrough (NMD candidate) [Source:HGNC Symbol;Acc:HGNC:49200]	N/A
VSTM5	V-set and transmembrane domain containing 5 [Source:HGNC Symbol;Acc:HGNC:34443]	N/A
VSTM1	V-set and transmembrane domain containing 1 [Source:HGNC Symbol;Acc:HGNC:29455]	N/A
UFSP1	UFM1-specific peptidase 1 (inactive) [Source:HGNC Symbol;Acc:HGNC:33821]	N/A
ZNF385C	zinc finger protein 385C [Source:HGNC Symbol;Acc:HGNC:33722]	N/A
RGPD8	RANBP2-like and GRIP domain containing 8 [Source:HGNC Symbol;Acc:HGNC:9849]	N/A
FAM182B	family with sequence similarity 182 member B [Source:HGNC Symbol;Acc:HGNC:34503]	N/A
GLIPR1L2	GLI pathogenesis-related 1 like 2 [Source:HGNC Symbol;Acc:HGNC:28592]	N/A
KIAA1143	KIAA1143 [Source:HGNC Symbol;Acc:HGNC:29198]	N/A
ARHGEF35	Rho guanine nucleotide exchange factor 35 [Source:HGNC Symbol;Acc:HGNC:33846]	N/A
NUPR2	nuclear protein 2, transcriptional regulator [Source:HGNC Symbol;Acc:HGNC:44164]	N/A
C8orf46	chromosome 8 open reading frame 46 [Source:HGNC Symbol;Acc:HGNC:28498]	N/A
FAM101A	family with sequence similarity 101 member A [Source:HGNC Symbol;Acc:HGNC:27051]	N/A
LY6G5B	lymphocyte antigen 6 complex, locus G5B [Source:HGNC Symbol;Acc:HGNC:13931]	N/A
C15orf40	chromosome 15 open reading frame 40 [Source:HGNC Symbol;Acc:HGNC:28443]	N/A
NEIL2	nei like DNA glycosylase 2 [Source:HGNC Symbol;Acc:HGNC:18956]	N/A
UMAD1	UBAP1-MVB12-associated (UMA) domain containing 1 [Source:HGNC Symbol;Acc:HGNC:48955]	N/A
CCDC36	coiled-coil domain containing 36 [Source:HGNC Symbol;Acc:HGNC:	N/A

Gene	Description	Rank
	27945]	
ZNF285	zinc finger protein 285 [Source:HGNC Symbol;Acc:HGNC:13079]	N/A
MPV17L	MPV17 mitochondrial inner membrane protein like [Source:HGNC Symbol;Acc:HGNC:26827]	N/A
FAM117B	family with sequence similarity 117 member B [Source:HGNC Symbol;Acc:HGNC:14440]	N/A
LY6K	lymphocyte antigen 6 complex, locus K [Source:HGNC Symbol;Acc:HGNC:24225]	N/A
TTC32	tetratricopeptide repeat domain 32 [Source:HGNC Symbol;Acc:HGNC:32954]	N/A
SMN2	survival of motor neuron 2, centromeric [Source:HGNC Symbol;Acc:HGNC:11118]	N/A
CCDC50	coiled-coil domain containing 50 [Source:HGNC Symbol;Acc:HGNC:18111]	N/A
CYP4V2	cytochrome P450 family 4 subfamily V member 2 [Source:HGNC Symbol;Acc:HGNC:23198]	N/A
KY	kyphoscoliosis peptidase [Source:HGNC Symbol;Acc:HGNC:26576]	N/A
ZNF468	zinc finger protein 468 [Source:HGNC Symbol;Acc:HGNC:33105]	N/A
EPHA6	EPH receptor A6 [Source:HGNC Symbol;Acc:HGNC:19296]	N/A
SIGLEC12	sialic acid binding Ig like lectin 12 (gene/pseudogene) [Source:HGNC Symbol;Acc:HGNC:15482]	N/A
MB21D2	Mab-21 domain containing 2 [Source:HGNC Symbol;Acc:HGNC:30438]	N/A
ANGPTL5	angiopoietin like 5 [Source:HGNC Symbol;Acc:HGNC:19705]	N/A
ZNF100	zinc finger protein 100 [Source:HGNC Symbol;Acc:HGNC:12880]	N/A
EXOC4	exocyst complex component 4 [Source:HGNC Symbol;Acc:HGNC:30389]	N/A
FAM167A	family with sequence similarity 167 member A [Source:HGNC Symbol;Acc:HGNC:15549]	N/A
CNTNAP4	contactin associated protein like 4 [Source:HGNC Symbol;Acc:HGNC:18747]	N/A
TCF19	transcription factor 19 [Source:HGNC Symbol;Acc:HGNC:11629]	N/A
ZNF749	zinc finger protein 749 [Source:HGNC Symbol;Acc:HGNC:32783]	N/A
MYCBPAP	MYCBP associated protein [Source:HGNC Symbol;Acc:HGNC:19677]	N/A
CCDC40	coiled-coil domain containing 40 [Source:HGNC Symbol;Acc:HGNC:26090]	N/A

Gene	Description	Rank
SMDT1	single-pass membrane protein with aspartate rich tail 1 [Source: HGNC Symbol;Acc:HGNC:25055]	N/A
SLC22A10	solute carrier family 22 member 10 [Source:HGNC Symbol;Acc: HGNC:18057]	N/A
FUT11	fucosyltransferase 11 [Source:HGNC Symbol;Acc:HGNC:19233]	N/A
LCA5L	LCA5L, lebercilin like [Source:HGNC Symbol;Acc:HGNC:1255]	N/A
PIDD1	p53-induced death domain protein 1 [Source:HGNC Symbol;Acc: HGNC:16491]	N/A
P4HA2	prolyl 4-hydroxylase subunit alpha 2 [Source:HGNC Symbol;Acc: HGNC:8547]	N/A
DCLK2	doublecortin like kinase 2 [Source:HGNC Symbol;Acc:HGNC:19002]	N/A
AS3MT	arsenite methyltransferase [Source:HGNC Symbol;Acc:HGNC:17452]	N/A
STK33	serine/threonine kinase 33 [Source:HGNC Symbol;Acc:HGNC:14568]	N/A
CCDC134	coiled-coil domain containing 134 [Source:HGNC Symbol;Acc:HGNC: 26185]	N/A
PSORS1C2	psoriasis susceptibility 1 candidate 2 [Source:HGNC Symbol;Acc: HGNC:17199]	N/A
SCRN2	secernin 2 [Source:HGNC Symbol;Acc:HGNC:30381]	N/A
MRPL10	mitochondrial ribosomal protein L10 [Source:HGNC Symbol;Acc: HGNC:14055]	N/A
SPATC1L	spermatogenesis and centriole associated 1-like [Source:HGNC Symbol;Acc:HGNC:1298]	N/A
MTHFS	5,10-methenyltetrahydrofolate synthetase (5-formyltetrahydrofolate cyclo-ligase) [Source:HGNC Symbol;Acc:HGNC:7437]	N/A
AKIP1	A-kinase interacting protein 1 [Source:HGNC Symbol;Acc:HGNC: 1170]	N/A
MRI1	methylthioribose-1-phosphate isomerase 1 [Source:HGNC Symbol; Acc:HGNC:28469]	N/A
OXGR1	oxoglutarate receptor 1 [Source:HGNC Symbol;Acc:HGNC:4531]	N/A
ZNF502	zinc finger protein 502 [Source:HGNC Symbol;Acc:HGNC:23718]	N/A
OSCAR	osteoclast associated, immunoglobulin-like receptor [Source:HGNC Symbol;Acc:HGNC:29960]	N/A
DRICH1	aspartate rich 1 [Source:HGNC Symbol;Acc:HGNC:28031]	N/A
NDUFV3	NADH:ubiquinone oxidoreductase subunit V3 [Source:HGNC Symbol;Acc:HGNC:7719]	N/A
TMEM209	transmembrane protein 209 [Source:HGNC Symbol;Acc:HGNC:	N/A

Gene	Description	Rank
	21898]	
BORCS7	BLOC-1 related complex subunit 7 [Source:HGNC Symbol;Acc:HGNC:23516]	N/A
COMMD2	COMM domain containing 2 [Source:HGNC Symbol;Acc:HGNC:24993]	N/A
CWF19L2	CWF19-like 2, cell cycle control (S. pombe) [Source:HGNC Symbol;Acc:HGNC:26508]	N/A
ZNF215	zinc finger protein 215 [Source:HGNC Symbol;Acc:HGNC:13007]	N/A
PSPH	phosphoserine phosphatase [Source:HGNC Symbol;Acc:HGNC:9577]	N/A
RHD	Rh blood group D antigen [Source:HGNC Symbol;Acc:HGNC:10009]	N/A
DCT	dopachrome tautomerase [Source:HGNC Symbol;Acc:HGNC:2709]	N/A
OR5P2	olfactory receptor family 5 subfamily P member 2 [Source:HGNC Symbol;Acc:HGNC:14783]	N/A
MSRA	methionine sulfoxide reductase A [Source:HGNC Symbol;Acc:HGNC:7377]	N/A
GRIK2	glutamate ionotropic receptor kainate type subunit 2 [Source:HGNC Symbol;Acc:HGNC:4580]	N/A
TMEM98	transmembrane protein 98 [Source:HGNC Symbol;Acc:HGNC:24529]	N/A
GLCCI1	glucocorticoid induced 1 [Source:HGNC Symbol;Acc:HGNC:18713]	N/A
RASAL1	RAS protein activator like 1 [Source:HGNC Symbol;Acc:HGNC:9873]	N/A
ZNF304	zinc finger protein 304 [Source:HGNC Symbol;Acc:HGNC:13505]	N/A
MRPS10	mitochondrial ribosomal protein S10 [Source:HGNC Symbol;Acc:HGNC:14502]	N/A
NICN1	nicolin 1 [Source:HGNC Symbol;Acc:HGNC:18317]	N/A
ART5	ADP-ribosyltransferase 5 [Source:HGNC Symbol;Acc:HGNC:24049]	N/A
IP6K2	inositol hexakisphosphate kinase 2 [Source:HGNC Symbol;Acc:HGNC:17313]	N/A
ACSM1	acyl-CoA synthetase medium-chain family member 1 [Source:HGNC Symbol;Acc:HGNC:18049]	N/A
MXRA7	matrix remodeling associated 7 [Source:HGNC Symbol;Acc:HGNC:7541]	N/A
NT5C3B	5'-nucleotidase, cytosolic IIIB [Source:HGNC Symbol;Acc:HGNC:28300]	N/A
THEM6	thioesterase superfamily member 6 [Source:HGNC Symbol;Acc:HGNC:29656]	N/A
HYAL3	hyaluronoglucosaminidase 3 [Source:HGNC Symbol;Acc:HGNC:5322]	N/A

Gene	Description	Rank
RANBP17	RAN binding protein 17 [Source:HGNC Symbol;Acc:HGNC:14428]	N/A
NUDT13	nudix hydrolase 13 [Source:HGNC Symbol;Acc:HGNC:18827]	N/A
ABO	ABO blood group (transferase A, alpha 1-3-N-acetylgalactosaminyltransferase; transferase B, alpha 1-3-galactosyltransferase) [Source:HGNC Symbol;Acc:HGNC:79]	N/A
OSER1	oxidative stress responsive serine rich 1 [Source:HGNC Symbol;Acc:HGNC:16105]	N/A
ZNF223	zinc finger protein 223 [Source:HGNC Symbol;Acc:HGNC:13016]	N/A
PSCA	prostate stem cell antigen [Source:HGNC Symbol;Acc:HGNC:9500]	N/A
TM7SF3	transmembrane 7 superfamily member 3 [Source:HGNC Symbol;Acc:HGNC:23049]	N/A
LY75	lymphocyte antigen 75 [Source:HGNC Symbol;Acc:HGNC:6729]	N/A
HHAT	hedgehog acyltransferase [Source:HGNC Symbol;Acc:HGNC:18270]	N/A
TIMM10	translocase of inner mitochondrial membrane 10 homolog (yeast) [Source:HGNC Symbol;Acc:HGNC:11814]	N/A
VPS13A	vacuolar protein sorting 13 homolog A [Source:HGNC Symbol;Acc:HGNC:1908]	N/A
NME7	NME/NM23 family member 7 [Source:HGNC Symbol;Acc:HGNC:20461]	N/A
PRKAR1B	protein kinase cAMP-dependent type I regulatory subunit beta [Source:HGNC Symbol;Acc:HGNC:9390]	N/A
CYP2D6	cytochrome P450 family 2 subfamily D member 6 [Source:HGNC Symbol;Acc:HGNC:2625]	N/A
SAT2	spermidine/spermine N1-acetyltransferase family member 2 [Source:HGNC Symbol;Acc:HGNC:23160]	N/A
PPP1R14A	protein phosphatase 1 regulatory inhibitor subunit 14A [Source:HGNC Symbol;Acc:HGNC:14871]	N/A
TOR3A	torsin family 3 member A [Source:HGNC Symbol;Acc:HGNC:11997]	N/A
KLKB1	kallikrein B1 [Source:HGNC Symbol;Acc:HGNC:6371]	N/A
SYCE1L	synaptonemal complex central element protein 1 like [Source:HGNC Symbol;Acc:HGNC:37236]	N/A
EDEM3	ER degradation enhancing alpha-mannosidase like protein 3 [Source:HGNC Symbol;Acc:HGNC:16787]	N/A
MTFR1	mitochondrial fission regulator 1 [Source:HGNC Symbol;Acc:HGNC:29510]	N/A
TRIM9	tripartite motif containing 9 [Source:HGNC Symbol;Acc:HGNC:16288]	N/A

Gene	Description	Rank
ZNF197	zinc finger protein 197 [Source:HGNC Symbol;Acc:HGNC:12988]	N/A
MTG1	mitochondrial ribosome associated GTPase 1 [Source:HGNC Symbol; Acc:HGNC:32159]	N/A
CDK10	cyclin dependent kinase 10 [Source:HGNC Symbol;Acc:HGNC:1770]	N/A
KLK7	kallikrein related peptidase 7 [Source:HGNC Symbol;Acc:HGNC: 6368]	N/A
CSDC2	cold shock domain containing C2 [Source:HGNC Symbol;Acc:HGNC: 30359]	N/A
PPM1B	protein phosphatase, Mg2+/Mn2+ dependent 1B [Source:HGNC Symbol;Acc:HGNC:9276]	N/A
FBN2	fibrillin 2 [Source:HGNC Symbol;Acc:HGNC:3604]	N/A
POLR2L	polymerase (RNA) II subunit L [Source:HGNC Symbol;Acc:HGNC: 9199]	N/A
ANAPC13	anaphase promoting complex subunit 13 [Source:HGNC Symbol;Acc: HGNC:24540]	N/A
CLK4	CDC like kinase 4 [Source:HGNC Symbol;Acc:HGNC:13659]	N/A
LAMA3	laminin subunit alpha 3 [Source:HGNC Symbol;Acc:HGNC:6483]	N/A
MCFD2	multiple coagulation factor deficiency 2 [Source:HGNC Symbol;Acc: HGNC:18451]	N/A
SH3BGR	SH3 domain binding glutamate rich protein [Source:HGNC Symbol; Acc:HGNC:10822]	N/A
BTN3A2	butyrophilin subfamily 3 member A2 [Source:HGNC Symbol;Acc: HGNC:1139]	N/A
PPDPF	pancreatic progenitor cell differentiation and proliferation factor [Source:HGNC Symbol;Acc:HGNC:16142]	N/A
CASD1	CAS1 domain containing 1 [Source:HGNC Symbol;Acc:HGNC:16014]	N/A
UROD	uroporphyrinogen decarboxylase [Source:HGNC Symbol;Acc:HGNC: 12591]	N/A
TPBG	trophoblast glycoprotein [Source:HGNC Symbol;Acc:HGNC:12004]	N/A
DNAAF5	dynein (axonemal) assembly factor 5 [Source:HGNC Symbol;Acc: HGNC:26013]	N/A
PARVG	parvin gamma [Source:HGNC Symbol;Acc:HGNC:14654]	N/A
NCKIPSD	NCK interacting protein with SH3 domain [Source:HGNC Symbol; Acc:HGNC:15486]	N/A
FUBP1	far upstream element binding protein 1 [Source:HGNC Symbol;Acc: HGNC:4004]	N/A

Gene	Description	Rank
NAGA	alpha-N-acetylgalactosaminidase [Source:HGNC Symbol;Acc:HGNC:7631]	N/A
ADAM19	ADAM metallopeptidase domain 19 [Source:HGNC Symbol;Acc:HGNC:197]	N/A
SNX2	sorting nexin 2 [Source:HGNC Symbol;Acc:HGNC:11173]	N/A
CTSO	cathepsin O [Source:HGNC Symbol;Acc:HGNC:2542]	N/A
TMX1	thioredoxin related transmembrane protein 1 [Source:HGNC Symbol;Acc:HGNC:15487]	N/A
CCDC92	coiled-coil domain containing 92 [Source:HGNC Symbol;Acc:HGNC:29563]	N/A
CEP63	centrosomal protein 63 [Source:HGNC Symbol;Acc:HGNC:25815]	N/A
DARS2	aspartyl-tRNA synthetase 2, mitochondrial [Source:HGNC Symbol;Acc:HGNC:25538]	N/A
ZNF354A	zinc finger protein 354A [Source:HGNC Symbol;Acc:HGNC:11628]	N/A
CD68	CD68 molecule [Source:HGNC Symbol;Acc:HGNC:1693]	N/A
RP11-382A20.3	Hepatoma-derived growth factor-related protein 3 [Source:UniProtKB/Swiss-Prot;Acc:Q9Y3E1]	N/A
GLO1	glyoxalase I [Source:HGNC Symbol;Acc:HGNC:4323]	N/A
SURF1	surfeit 1 [Source:HGNC Symbol;Acc:HGNC:11474]	N/A
POLI	polymerase (DNA) iota [Source:HGNC Symbol;Acc:HGNC:9182]	N/A
ATP1B2	ATPase Na+/K+ transporting subunit beta 2 [Source:HGNC Symbol;Acc:HGNC:805]	N/A
TRIM66	tripartite motif containing 66 [Source:HGNC Symbol;Acc:HGNC:29005]	N/A
SYNM	synemin [Source:HGNC Symbol;Acc:HGNC:24466]	N/A
DOK5	docking protein 5 [Source:HGNC Symbol;Acc:HGNC:16173]	N/A
WDR45B	WD repeat domain 45B [Source:HGNC Symbol;Acc:HGNC:25072]	N/A
IMMT	inner membrane mitochondrial protein [Source:HGNC Symbol;Acc:HGNC:6047]	N/A
UBR2	ubiquitin protein ligase E3 component n-recognin 2 [Source:HGNC Symbol;Acc:HGNC:21289]	N/A
POU5F1	POU class 5 homeobox 1 [Source:HGNC Symbol;Acc:HGNC:9221]	N/A
NUBP1	nucleotide binding protein 1 [Source:HGNC Symbol;Acc:HGNC:8041]	N/A
WRB	tryptophan rich basic protein [Source:HGNC Symbol;Acc:HGNC:12790]	N/A
PPIP5K2	diphosphoinositol pentakisphosphate kinase 2 [Source:HGNC Symbol;	N/A

Gene	Description	Rank
	Acc:HGNC:29035]	
GGH	gamma-glutamyl hydrolase [Source:HGNC Symbol;Acc:HGNC:4248]	N/A
CAAP1	caspase activity and apoptosis inhibitor 1 [Source:HGNC Symbol; Acc:HGNC:25834]	N/A
SKIV2L	Ski2 like RNA helicase [Source:HGNC Symbol;Acc:HGNC:10898]	N/A
PBX3	PBX homeobox 3 [Source:HGNC Symbol;Acc:HGNC:8634]	N/A
CASP7	caspase 7 [Source:HGNC Symbol;Acc:HGNC:1508]	N/A
YWHAE	tyrosine 3-monooxygenase/tryptophan 5-monooxygenase activation protein epsilon [Source:HGNC Symbol;Acc:HGNC:12851]	N/A
RBM6	RNA binding motif protein 6 [Source:HGNC Symbol;Acc:HGNC: 9903]	N/A
ATIC	5-aminoimidazole-4-carboxamide ribonucleotide formyltransferase/ IMP cyclohydrolase [Source:HGNC Symbol;Acc:HGNC:794]	N/A
TPO	thyroid peroxidase [Source:HGNC Symbol;Acc:HGNC:12015]	N/A
BARD1	BRCA1 associated RING domain 1 [Source:HGNC Symbol;Acc: HGNC:952]	N/A
HLA-B	major histocompatibility complex, class I, B [Source:HGNC Symbol; Acc:HGNC:4932]	N/A
HLA-DQB2	major histocompatibility complex, class II, DQ beta 2 [Source:HGNC Symbol;Acc:HGNC:4945]	N/A
HLA-DOB	major histocompatibility complex, class II, DO beta [Source:HGNC Symbol;Acc:HGNC:4937]	N/A
HLA-DQA2	major histocompatibility complex, class II, DQ alpha 2 [Source: HGNC Symbol;Acc:HGNC:4943]	N/A
HLA-DRB5	major histocompatibility complex, class II, DR beta 5 [Source:HGNC Symbol;Acc:HGNC:4953]	N/A
HLA-DQB1	major histocompatibility complex, class II, DQ beta 1 [Source:HGNC Symbol;Acc:HGNC:4944]	N/A
HLA-DRB1	major histocompatibility complex, class II, DR beta 1 [Source:HGNC Symbol;Acc:HGNC:4948]	N/A
HLA-DRB3	major histocompatibility complex, class II, DR beta 3 [Source:HGNC Symbol;Acc:HGNC:4951]	1
HLA-DRB4	major histocompatibility complex, class II, DR beta 4 [Source:HGNC Symbol;Acc:HGNC:4952]	2
HLA-DPB1	major histocompatibility complex, class II, DP beta 1 [Source:HGNC Symbol;Acc:HGNC:4940]	3
HLA-DMB	major histocompatibility complex, class II, DM beta [Source:HGNC	4

Gene	Description	Rank
	Symbol;Acc:HGNC:4935]	
XXbac-BPG246D15.9		5
HLA-DQA1	major histocompatibility complex, class II, DQ alpha 1 [Source:HGNC Symbol;Acc:HGNC:4942]	6
HLA-DRA	major histocompatibility complex, class II, DR alpha [Source:HGNC Symbol;Acc:HGNC:4947]	7
HLA-DPA1	major histocompatibility complex, class II, DP alpha 1 [Source:HGNC Symbol;Acc:HGNC:4938]	8
XXbac-BPG181M17.5		9
HLA-DMA	major histocompatibility complex, class II, DM alpha [Source:HGNC Symbol;Acc:HGNC:4934]	10
HLA-DOA	major histocompatibility complex, class II, DO alpha [Source:HGNC Symbol;Acc:HGNC:4936]	11
HLA-F	major histocompatibility complex, class I, F [Source:HGNC Symbol;Acc:HGNC:4963]	12
HLA-C	major histocompatibility complex, class I, C [Source:HGNC Symbol;Acc:HGNC:4933]	13
HLA-A	major histocompatibility complex, class I, A [Source:HGNC Symbol;Acc:HGNC:4931]	14
HLA-G	major histocompatibility complex, class I, G [Source:HGNC Symbol;Acc:HGNC:4964]	15
HLA-E	major histocompatibility complex, class I, E [Source:HGNC Symbol;Acc:HGNC:4962]	16
CD3E	CD3e molecule [Source:HGNC Symbol;Acc:HGNC:1674]	17
CD4	CD4 molecule [Source:HGNC Symbol;Acc:HGNC:1678]	18
LCK	LCK proto-oncogene, Src family tyrosine kinase [Source:HGNC Symbol;Acc:HGNC:6524]	19
CD3G	CD3g molecule [Source:HGNC Symbol;Acc:HGNC:1675]	20
CD3D	CD3d molecule [Source:HGNC Symbol;Acc:HGNC:1673]	21
HFE	hemochromatosis [Source:HGNC Symbol;Acc:HGNC:4886]	22
MR1	major histocompatibility complex, class I-related [Source:HGNC Symbol;Acc:HGNC:4975]	23
FCGRT	Fc fragment of IgG receptor and transporter [Source:HGNC Symbol;Acc:HGNC:3621]	24
AZGP1	alpha-2-glycoprotein 1, zinc-binding [Source:HGNC Symbol;Acc:	25

Gene	Description	Rank
	HGNC:910]	
GZMB	granzyme B [Source:HGNC Symbol;Acc:HGNC:4709]	26
PRF1	perforin 1 [Source:HGNC Symbol;Acc:HGNC:9360]	27
B2M	beta-2-microglobulin [Source:HGNC Symbol;Acc:HGNC:914]	28
CD80	CD80 molecule [Source:HGNC Symbol;Acc:HGNC:1700]	29
FAS	Fas cell surface death receptor [Source:HGNC Symbol;Acc:HGNC: 11920]	30

Networks

**Consolidated-Pathways-2013**	**46.15%**
Autoimmune thyroid disease	6.74%
Translocation of ZAP-70 to ...	6.60%
Phosphorylation of CD3 and ...	5.39%
PD-1 signaling	4.93%
Allograft rejection	4.19%
Graft-versus-host disease	3.92%
Type I diabetes mellitus	3.60%
Asthma	3.07%
Interferon gamma signaling	2.97%
Generation of second messenger ...	2.80%
Downstream TCR signaling	1.93%

**InterPro**	**36.58%**
MHC_II_b_N	16.44%
Ig/MHC_CS	7.26%
MHC_I/II-like_Ag-recog	6.82%
Ig_C1-set	6.06%

**Co-expression**	**13.20%**
Mallon-McKay-2013<br>StemCellDB: the human pluripotent stem cell database at the National Institutes of Health. Mallon et al (2013). *Stem Cell Res*<br>Co-expression with 585,265 interactions from GEO	2.25%
Chen-Brown-2002<br>Gene expression patterns in human liver cancers. Chen et al (2002). *Mol Biol Cell*<br>Co-expression with 282,241 interactions from supplementary material	1.62%
Noble-Diehl-2008<br>Regional variation in gene expression in the healthy colon is dysregulated in ulcerative colitis. Noble et al (2008). *Gut*<br>Co-expression with 661,539 interactions from GEO	1.60%
Rosenwald-Staudt-2001<br>Relation of gene expression phenotype to immunoglobulin mutation genotype in B cell chronic lymphocytic leukemia. Rosenwald et al (2001). *J Exp Med*<br>Co-expression with 114,694 interactions from supplementary material	1.25%

Co-expression 13.20%

Wang-Cheung-2015 1.17%

Genetic variation in insulin-induced kinase signaling. Wang et al (2015). *Mol Syst Biol*

Co-expression with 411,047 interactions from GEO

Alizadeh-Staudt-2000 0.90%

Distinct types of diffuse large B-cell lymphoma identified by gene expression profiling. Alizadeh et al (2000). *Nature*

Co-expression with 90,336 interactions from supplementary material

Burington-Shaughnessy-2008 0.89%

Tumor cell gene expression changes following short-term in vivo exposure to single agent chemotherapeutics are related to survival in multiple myeloma. Burington et al (2008). *Clin Cancer Res*

Co-expression with 290,538 interactions from GEO

Smirnov-Cheung-2009 0.87%

Genetic analysis of radiation-induced changes in human gene expression. Smirnov et al (2009). *Nature*

Co-expression with 461,500 interactions from GEO

Bahr-Bowler-2013 0.71%

Peripheral blood mononuclear cell gene expression in chronic obstructive pulmonary disease. Bahr et al (2013). *Am J Respir Cell Mol Biol*

Co-expression with 274,949 interactions from GEO

Perou-Botstein-2000 0.52%

Molecular portraits of human breast tumours. Perou et al (2000). *Nature*

Co-expression with 185,068 interactions from supplementary material

Bild-Nevins-2006 B 0.49%

Oncogenic pathway signatures in human cancers as a guide to targeted therapies. Bild et al (2006). *Nature*

Co-expression with 280,683 interactions from GEO

Innocenti-Brown-2011 0.43%

Identification, replication, and functional fine-mapping of expression quantitative trait loci in primary human liver tissue. Innocenti et al (2011). *PLoS Genet*

Co-expression with 603,765 interactions from GEO

Wu-Garvey-2007 0.36%

The effect of insulin on expression of genes and biochemical pathways in human skeletal muscle. Wu et al (2007). *Endocrine*

Co-expression with 267,109 interactions from GEO

Dobbin-Giordano-2005 0.11%

Interlaboratory comparability study of cancer gene expression analysis using oligonucleotide microarrays. Dobbin et al (2005). *Clin Cancer Res*

Co-expression with 444,931 interactions from GEO

Wang-Maris-2006 0.03%

Integrative genomics identifies distinct molecular classes of neuroblastoma and shows that multiple genes are targeted by regional alterations in DNA copy number. Wang et al (2006). *Cancer Res*

Co-expression with 264,023 interactions from GEO

Physical Interactions 3.49%

Camargo-Brandon-2007 1.55%

Physical Interactions 3.49%

Camargo-Brandon-2007

Disrupted in Schizophrenia 1 Interactome: evidence for the close connectivity of risk genes and a potential synaptic basis for schizophrenia. Camargo et al (2007). *Mol Psychiatry*

Physical Interactions with 267 interactions from iRefIndex

Woods-Monteiro-2012 0.62%

Charting the landscape of tandem BRCT domain-mediated protein interactions. Woods et al (2012). *Sci Signal*

Physical Interactions with 919 interactions from iRefIndex

Oliviero-Cagney-2015 0.60%

The variant Polycomb Repressor Complex 1 component PCGF1 interacts with a pluripotency sub-network that includes DPPA4, a regulator of embryogenesis. Oliviero et al (2015). *Sci Rep*

Physical Interactions with 675 interactions from BioGRID

Huttlin-Gygi-2015 0.45%

The BioPlex Network: A Systematic Exploration of the Human Interactome. Huttlin et al (2015). *Cell*

Physical Interactions with 23,399 interactions from BioGRID

IREF-HPRD 0.20%

Physical Interactions with 34,206 interactions from iRefIndex

IREF-SMALL-SCALE-STUDIES 0.07%

Physical Interactions with 71,996 interactions from iRefIndex

Shared protein domains 0.40%

INTERPRO 0.40%

Shared protein domains with 608,863 interactions from InterPro

Predicted 0.19%

Stuart-Kim-2003 0.19%

A gene-coexpression network for global discovery of conserved genetic modules. Stuart et al (2003). *Science*

Predicted with 24,872 interactions from supplementary material
